# Revealing the Role of Self-Assembly Behavior of High-Assembly-Index Nano Amylopectin Ternary Complexes in the Slow Digestion Mechanism

**DOI:** 10.3390/foods15010002

**Published:** 2025-12-19

**Authors:** Bo Li, Chongxing Huang, Weihong Lu, Xin Yang

**Affiliations:** 1 School of Medicine and Health, Harbin Institute of Technology, Harbin 150001, China; 13654529477@163.com; 2 College of Light Industry and Food Engineering, Guangxi University, Nanning 530003, China; huangcx21@163.com

**Keywords:** nano endogenous amylopectin ternary complex, high self-assembly index complex, self-assembly process, multi-scale structure, log-of-slope digestive kinetics

## Abstract

Starch complexes have recently been identified as a new dietary supplement for dietary intervention in glycemic metabolism disorders. However, although the amylopectin significantly influenced starch complexes’ anti-digestibility, the underlying regulatory pattern remains unclear. Accordingly, this study constructed nano white waxy maize amylopectin (WMA) ternary complexes with a high self-assembly index (SI, 82.58%) using an ultrasound-assisted approach. And the relationship between self-assembly behavior and slow digestibility was revealed. Combined analyses of chemometrics revealed that during the WMA ternary self-assembly process, the increasing free side chains and α-1,6 glycosidic linkages contributed to the rise in potential, thereby generating more assembly sites and binding energy and ultimately elevating SI. Then, along with the transition from a diffuse state to V_h_-type crystallinity and spherical configuration, increases in relative crystallinity, double helices, molecular weight, short-range order, and gel-network viscous were observed, whereas semicrystalline lamellar thickness and “blocklet” size decreased. These indicated that both the number and dimensions of hydrolysis channels were reduced. Consequently, the increasing gelatinization temperature led to rising slowly digestible starch content (19.86–43.28%), causing a more stable glycemic release after WMA ternary self-assembly. This investigation provides a key theoretical and technological foundation for the development of novel slow-digesting precision nutrition ingredients.

## 1. Introduction

**Figure 1:**
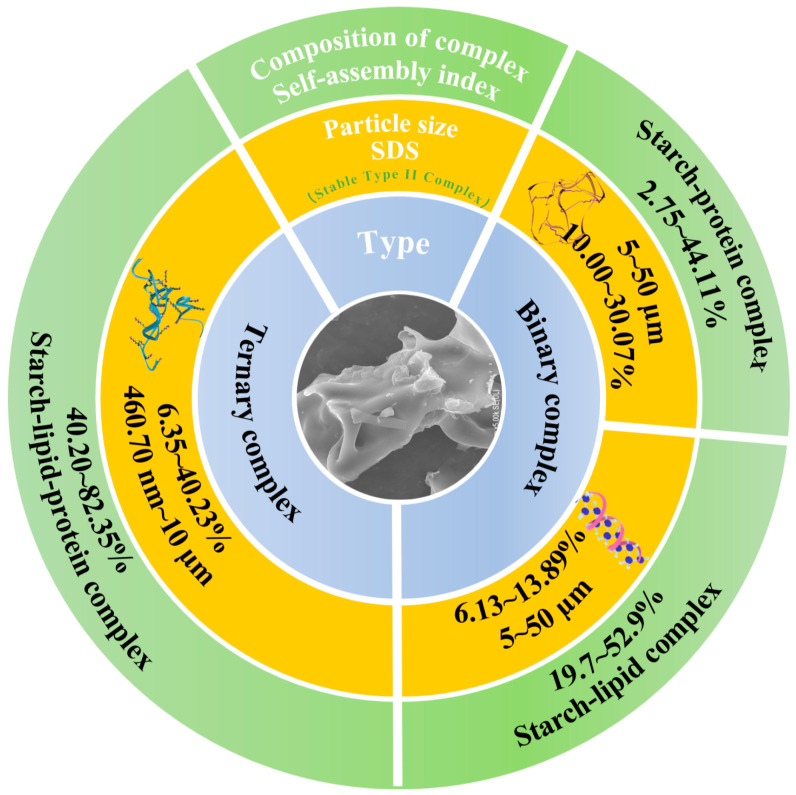
The comparison of slow digestibility, SI, and particle size between high-SI starch ternary complex and binary complex [1,2,3,4,5,6,8,9,10,11,12,13].

Starch, lipid, and protein constitute the principal macronutrient classes that underpin human dietary intake. Starch is a semicrystalline supramolecule composed of amylose (20–30%) and amylopectin (70–80%) [1]. The side chains of amylopectin can significantly determine the multi-scale supramolecular structure [2]. Notably, waxy starch is characterized by an exceptionally high amylopectin content (exceeding 99%). And amylose, predominantly located in amorphous regions, can be rapidly hydrolyzed, whereas amylopectin, mainly distributed in densely packed crystalline regions, hinders the penetration of digestive enzymes [3]. Based on this, waxy starch provides a structural basis that facilitates the development of resistant starch (RS) or slowly digestible starch (SDS) [4,5]. While RS offers health benefits such as attenuating postprandial glycemia, the extremely limited glycemia release restricts its nutritional value [6]. In contrast, SDS provides a more balanced digestive profile, releasing glycemia slowly. The SDS content also has a highly significant effect on starch log-of-slope (LOS) digestion kinetics, which can be used to estimate multi-scale (≥2 levels) kinetic constants and estimated glycemic index (EGI) [7]. Moreover, starch granules extracted on an industrial scale often have residual lipids and protein on their surface. Upon thermal processing, starch can self-assemble with lipid and protein on the starch granule surface through non-covalent bonds, markedly altering the SDS value [4]. According to their compositional characteristics, starch complexes were generally classified into binary complexes and ternary assemblies [2]. In contrast to binary systems, the formation of high self-assembly index (SI) starch ternary complexes involved an electrostatically induced hyperconjugative effect that originated from the polar head and tail groups of the protein molecules [1,2,3]. This phenomenon intensified and expanded the interaction forces that governed the assembly among starch chains, lipid chains, and the amide II region of the protein [4,5,6]. As a result, the ternary complexes developed a more compact and structurally ordered configuration, accompanied by a substantial increase in the assembly index [8,9,10]. The granular domains became densely organized, numerous enzymatic access sites were effectively shielded, and the overall resistance to enzymatic hydrolysis was markedly reinforced [11,12,13]. As illustrated in Figure 1, the proportions of slowly digestible and digestion-resistant fractions in the ternary complexes were considerably higher than those observed in the binary counterparts. However, the dynamic changes in multi-scale molecular structures, SDS content, and LOS digestion kinetics during amylopectin ternary self-assembly have rarely been reported.

Current research has mostly focused on the investigation of digestive mechanisms for amylose ternary complexes, such as by altering the degree of polymerization (Dp) [2], the amylose content [8], and ductility [6,8]. By comparison, research on amylopectin complexes is still scarce. Wang et al. [1,9] found that although the self-assembly index (SI) of amylopectin–lipid or amylopectin–protein complexes is markedly lower than that of amylose complexes, both show comparable digestibility. Previous research [2,10] reported that lipids can bind with amylopectin long side chains, significantly enhancing resistance to enzymatic hydrolysis compared with native starch. Zhao et al. [11] demonstrated that amylopectin and protein can generate denser non-covalent complexes through self-assembly behavior, which increases protein β-sheet content and reduces enzymatic permeability to nano surfaces. Yao et al. [7] inferred that, for the low-RS materials, interactions between protein and lipid might induce hyperconjugation effects, thereby promoting amylopectin short-chain migration or degradation and facilitating limited amylopectin ternary complex formation. Li et al. [4] confirmed the existence of amylopectin–lipid–protein complex molecules with higher SDS content, while its lower SI (≤50%) and larger particle size compared to amylose complexes limited its further development and utilization. Other previous research found that organic reagent heat-induced self-assembly [8], enzymatic degradation [6], and ultrasonication treatment [12] could reduce the average diameter of amylose-based complexes and improve their SI. Among them, ultrasonication technology is an effective one without the addition of chemical reagents. And the middle–high power ultrasonication technology within 140–640 W at 20 kHz for 30–60 min could effectively improve the particle aggregation and facilitate the formation of nanoparticles, without disrupting the internal structure of the amylose complexes [13]. Compared with physical modification techniques involving high pressure, high temperature, and intense shear, as well as chemical modification methods [12]. This structural compaction strengthens both short-range and long-range order, contributes to a marked reduction in particle size, and produces granules with higher resistance to thermal and enzymatic disruption [2]. Overall, the ultrasound treatment used in this study served as an effective auxiliary approach, preserving the double-helical structure and crystalline order without causing structural damage, while enabling the complexes to reach nanoscale particle size and improving their particle integrity [2,13]. Meanwhile, evidence remains limited on the role of self-assembly behavior of high-SI nano amylopectin ternary complexes, particularly those treated with medium–high power ultrasonication, in the slow digestion mechanism.

Based on these, a high-SI nano white waxy maize amylopectin (WMA) endogenous ternary complex was made by response surface methodology and ultrasonication treatment. WMA paste, WMA–endogenous protein complex, and WMA–endogenous lipid complex were prepared for comparative analysis. The changes in multi-scale structure during WMA endogenous ternary self-assembly were systematically revealed through self-assembly properties, glycosidic bond structures, helical conformations, gelatinization characteristics, and spatial structural characteristics. Subsequently, digestible fractions and LOS digestion kinetics were characterized to verify slow-digestion performance upon ternary self-assembly. Chemometric analysis was employed to clarify the role of the self-assembly behavior of amylopectin ternary complexes in the slow digestion mechanism. This research innovatively clarified the self-assembly driving mode of the WMA ternary complex by first analyzing its fine structural characteristics and then resolving the subsequent hierarchical structures in sequence, ultimately establishing their connection to the emergence of slow digestibility. This investigation supplements current research on the self-assembly mechanism of nanoscale amylopectin ternary complexes, while providing theoretical guidance for the development of functional foods targeting hyperglycemia prevention and a foundation for the high-value utilization of white waxy maize.

## 2. Materials and Methods

### 2.1. Materials

A local agricultural market in Harbin, China, served as the source of the white waxy maize used in this study. Amyloglucosidase (3300 U/mL), glucose oxidase–peroxidase (1000 U/mg), and α-amylase (100,000 U/g) were all purchased from Megazyme Ltd. (Bray, Ireland).

### 2.2. Amylopectin Isolation

Waxy maize flour was dispersed and homogenized in distilled water at a ratio of 1:3 (*w*/*w*) using a colloid mill to prepare a starch slurry. The slurry was subsequently filtered by a 200-mesh sieve, and the filtrate was subsequently centrifuged at 6000× *g* for 10 min. The obtained crude starch was then mixed with a 0.5 M sodium thiosulfate (*w*/*w* 1:1) until complete reaction and centrifuged (5000× *g*, 10 min). And the brown epidermal layer on the centrifuged precipitate was carefully scraped off to obtain white waxy maize amylopectin (WMA). WMA underwent triple rinsing with 50% aqueous ethanol, followed by drying (50 °C, 24 h). The detected amylopectin content was 99.84% [14].

### 2.3. Endogenous Protein and Lipid Isolation

The endogenous protein and lipid were isolated based on a method reported by Li et al. [4]. Mature maize kernels were dried, ground, and then homogenized with 5% ethanol at a 1:10 ratio (*w*/*w*). This slurry was then processed using a HA640–70–27–C II supercritical CO_2_ apparatus produced by Nantong Huaan Supercritical Extraction Co. (Nantong, China). Endogenous lipid was then isolated under conditions at 45 °C and 68.5 L/h. During the extraction, the extraction chamber (29 MPa) and the two sequential separation chambers (10 and 4 MPa) were used to purify. The lipid-rich fraction was collected and concentrated by rotary evaporation to remove residual solvent, yielding purified endogenous lipids.

For protein extraction, mature maize kernels were defatted twice with ethyl acetate (1:2, *w*/*w*). The lipid-free flour was dispersed in an alkaline solution at pH = 9.0 with a solid-to-liquid ratio of 1:10 (*w*/*w*) and separated by centrifugation (5000× *g*, 15 min). In total, 1 M HCl was used to adjust the pH of the resulting supernatant to 4.3 in order to induce isoelectric precipitation, after which the mixture was centrifuged (6000× *g*, 20 min). This precipitate was mixed with deionized water and subsequently purified by dialysis through a 7 kDa cutoff membrane, yielding a protein fraction composed primarily of globulins and albumins.

### 2.4. Construction of High-SI Nano Endogenous Amylopectin Ternary Complex

#### 2.4.1. Construction of Amylopectin Ternary Complex and Viscous Properties Analysis

A total of 2.0 g of WMA, 150–250 mg of endogenous lipids, and 100–200 mg of endogenous proteins was placed into a canister mounted on a temperature-controlled magnetic stirrer (IKA RCT 5). Deionized water was used to adjust the total weight of the mixture to 28.0 g. The slurry was first agitated at 960 rpm for 10 s and then maintained under steady mixing at 160 rpm during all following operations. It was subsequently brought to 50 °C (1 min), heated further to 95 °C (20 min), and afterwards lowered to 25 °C for a 10 min hold. The complex was dried at 60 °C until the moisture content was reduced to below 8%, washed with 50% ethanol, and subsequently freeze-dried [15]. WMA paste, WMA–endogenous lipid complexes, and WMA–endogenous protein complexes were prepared following the same procedure described above. And the viscous properties were evaluated through a Rapid Visco Analyzer (RVA) following the Standard 1 program [1].

#### 2.4.2. Preparation of Nano Amylopectin Ternary Complexes

WMA complexes were dispersed in distilled water at a ratio of 1:100 (*w*/*w*) and then processed under ultrasonic treatment at 600 W and 20 kHz for a total duration of 1 h (Cole-Parmer Instruments, Vernon Hills, IL, USA). The system operated in a pulsed power mode in which the probe was activated for 10 s, followed by a 10 s pause. A standard ultrasonic probe with a diameter of approximately 13 mm was used, and the vibration amplitude was maintained at 90 throughout the procedure. The acoustic energy density was 1.5 J/mL. The treatment was performed in a temperature-controlled vessel with continuous external cooling to prevent thermal accumulation. Following ultrasonication, the suspension was centrifuged at 3000× *g* for 15 min to collect the precipitate and then passed through an 800-mesh sieve, which was subsequently freeze-dried. It is noteworthy that ultrasonication markedly increased the SI [13].

#### 2.4.3. Response Surface Methodology for SI Optimization

Combined with the single-factor outcomes of WMA–lipid complex and WMA–protein complex, lipid and protein levels were selected as independent variables, and the self-assembly index (SI) of the ternary complex was used as the response variable. A two-factor, two-level response surface evaluation was carried out through a Box–Behnken scheme using Design-Expert 13 (Stat-Ease, Minneapolis, MN, USA), with specific analytical outcomes presented in the Results and Discussion Section.

### 2.5. SI Analysis

In total, 100 mg of starch complex was mixed with 10 mL of dissolution liquid containing ethanol and NaOH (1:9, *v*/*v*), followed by a 10 min heating step in boiling water. After being brought back to 25 °C, the preparation was adjusted to a final volume of 100 mL. From this solution, 2.5 mL was withdrawn and expanded to 50 mL, after which 1 mL of Lugol’s iodine solution was added and allowed to react for 20 min to generate color. The optical density at 548 nm was then obtained using a UV–visible instrument, and an amylopectin standard was used to construct the calibration curve [1]. The SI was calculated as follows.

(1) $$SI(\%)=100\times \left({Absorbance}_{native\;starch}-{Absorbance}_{comnplexes}\right)/{Absorbance}_{native\;starch}$$

### 2.6. Supramolecular Structure Investigation

#### 2.6.1. One-Dimensional and Two-Dimensional FTIR Analysis

The Hilbert transform algorithm was applied to analyze the characteristic peaks of the complexes within the range of 4000–400 cm^−1^ in 2D correlation spectroscopy (2D-COS) using a Nicolet 6700 FTIR system (Thermo Fisher Scientific, Waltham, MA, USA). The WMA paste was the reference, and the complexes were the stimulus response variables. The FTIR band-intensity ratio at 1047 to 1022 cm^−1^ was used to quantify the short-range order [16].

#### 2.6.2. Semicrystalline Lamella Structure

The samples were mixed with water at a ratio of 1:10 (*w*/*w*) to form a non-Newtonian fluid and then centrifuged at 6000 *g* for 15 min. The supernatant was transferred into the sample cell and analyzed using a 2D small-angle X-ray scattering instrument (SAXS, NanoSTAR, Bruker AXS Inc.,Billerica, Massachusetts, USA) with Cu Kα radiation at 50 kV and 30 W and a wavelength of 1.5418 Å. The scanning range is 0.010 < *q* < 0.25 Å^−1^, and the pure water background was subtracted [2]. The 2D patterns were then converted into 1D scattering curves, which were calculated by the calculation Formula (2) used to calculate the scattering space distance (*r*), scattering vector (*q*), and scattering intensity (*I*(*q*)). Moreover, the thickness parameters of semicrystalline lamellar (*d*), crystalline lamellar (*d_c_*), and amorphous lamellar (*d_a_*) of samples were obtained from the 1D curves. The surface fractal dimension (α) of the samples was calculated by *I(q)~q^a^*. And the mass fractal dimension (*Dm*) was analyzed by Formula (3) [17].

(2) $$L\left(r\right)=\frac{\int_{0}^{\infty }I\left(q\right)q^{2}\cos\left(qr\right)dq}{\int_{0}^{\infty }I\left(q\right)q^{2}dq}$$

(3) *Dm* = −*α* (−3 < *α* < −1)

#### 2.6.3. Crystalline Structure Analysis

The crystalline structure of the samples was analyzed using X-ray diffraction (XRD, 2θ = 4–40°, scan rate = 4°/min). JADE 6.5 software was employed to determine relative crystallinity (Rc) and crystal type [2].

#### 2.6.4. Zeta Potential and Particle Size Distribution Analysis

Since the starch complexes exhibited stronger hydrophobicity than the native starch, the particles of starch complexes rapidly aggregated when water was added, which hindered accurate particle size measurement [1]. Therefore, after dispersing 100 mg of the sample in 100 mL of distilled water, the suspension was sonicated under medium power (200 W) for 10 h to remove large-scale aggregation and ensure accurate particle size determination. The potential and particle size profile of the starch sample dispersion liquid was subsequently quantified by a nanoparticle size and Zeta potential analyzer [18,19].

#### 2.6.5. Helical Conformation Analysis

A starch–DMSO-d_6_ solution (1:100, *w*/*w*) was analyzed by 13C and 1H NMR spectroscopy with 9000 and 128 scans, respectively. The resulting spectra were processed using NMR built-in software (TopSpin 4.1) and Peakfit V4.12 to determine the content of glycosidic bonds and helical conformation [7].

#### 2.6.6. Molecular Configuration and Conformation

In total, 10 mg of starch paste and its complex samples were completely dissolved in 5 mL of DMSO containing 0.2% LiBr (50 mM, 90 °C, 24 h). The dissolution solution was diluted fourfold with DMSO/LiBr and subsequently examined using a size-exclusion chromatographic setup equipped with both light-scattering and refractive-index modules (Wyatt Technology, Santa Barbara, CA, USA). A 100 µL injection was introduced onto the Shodex OHpak SB-HQ Phenogel column (Showa Denko, Tokyo, Japan) at 60 °C with a flow rate of 0.3 mL/min. Data processing was conducted using the built-in software to extract the weight-average and number-average molar masses (Mw and Mn), the dispersity value (PI), the gyration radius (Rg), and molecular density index (*ρ*). The conformation index (ν) was derived by correlating molecular weight with the mean square radius of gyration [2].

#### 2.6.7. Nano Surface Texture Characteristics

**Figure A1:**
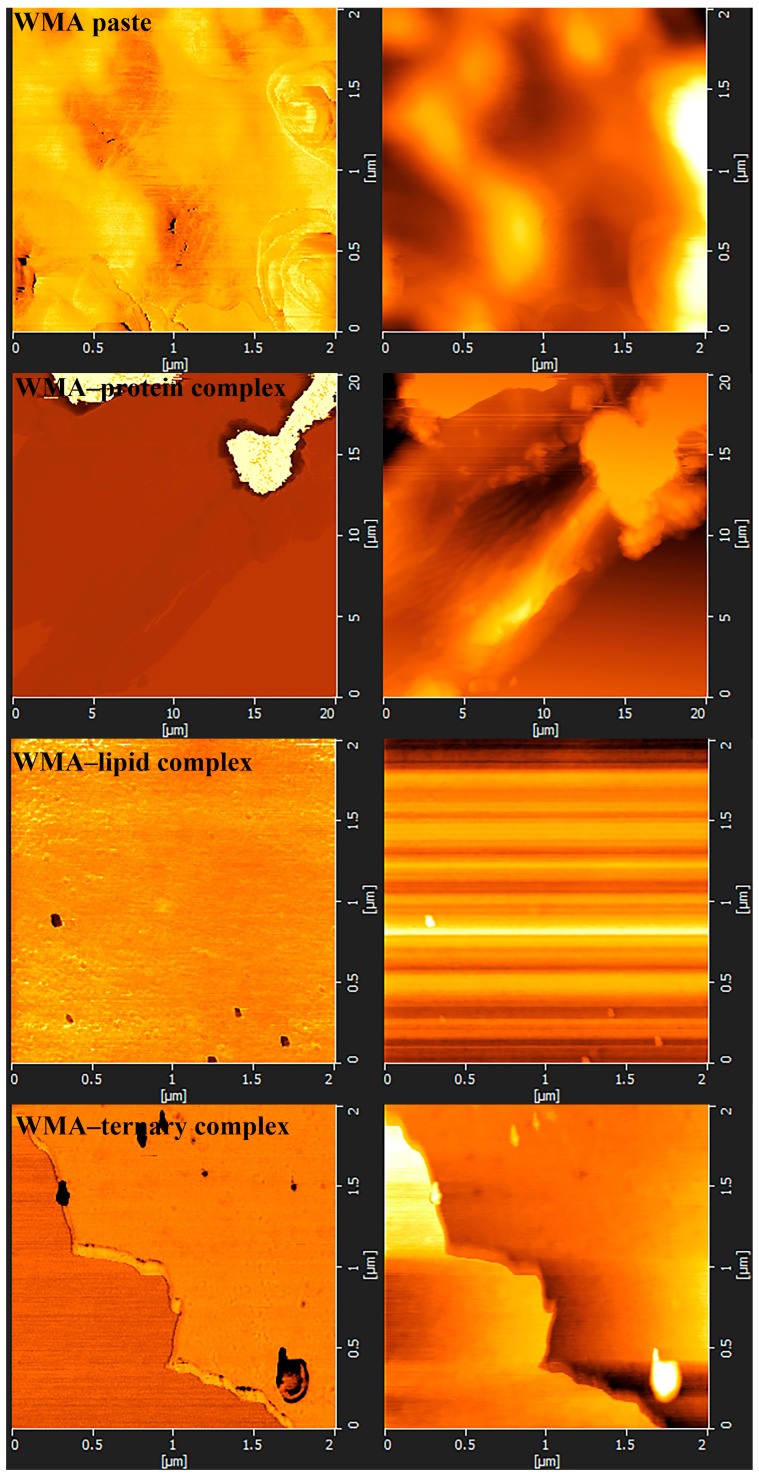
Nano surfaces, including phase image and stiffness map of samples.

Complex samples were scanned over a 2 μm × 2 μm area in tapping mode using an atomic force microscope (AFM, 5100N; Hitachi, Tokyo, Japan) to obtain phase images, modulus maps, and 3D surface profiles. The embedded software (PicoView 1.0) was used to calculate the root-mean-square roughness (Rq), which reflects the size of the “blocklet” protrusion structure [20]. In addition, energy, contrast, homogeneity, entropy, and fractal dimension were quantified from the phase images and stiffness maps (Figure A1) using the instrument PicoView 1.0.

#### 2.6.8. Micromorphology Analysis

The morphology features of sample granules were visualized with a high-resolution field-emission SEM instrument (SU8220, Hitachi, Japan) operated at 10 kV and viewed at 50,000× enlargement [19].

### 2.7. Gelatinization Properties

Starch paste and its complex sample slurries (1:3, *w*/*w*, with water) were placed in a liquid crucible and allowed to equilibrate for 12 h. Thermal transitions were subsequently characterized by differential scanning calorimetry (DSC) over a range of 10–120 °C (10 °C/min). And gelatinization parameters were analyzed, including the enthalpy change associated with gelatinization (ΔHg), as well as the peak (Tp), onset (To), end-point (Tc), and corresponding gelatinization interval temperature (R) [8].

### 2.8. Characterization of Slow Digestibility

#### 2.8.1. Digestive Fraction Analysis

WMA paste and its complexes samples (1 g) were suspended in a 10 mL digestive enzyme acetate buffer (0.1 mol/L sodium, pH = 5.2). Subsequently, 10 mL of an enzyme mixture containing amyloglucosidase (13 U/mL), invertase (190 U/mL), and α-amylase (3800 U/mL) was introduced. The preparation was maintained at 37 °C with orbital agitation at 180 rpm. At the digestion intervals of 20 and 120 min, 0.5 mL aliquots were withdrawn. In total, 20 mL of 70% ethanol was immediately mixed with the aliquots to halt the catalytic activity. After centrifugation (4000 rpm, 10 min), the glucose concentration in the enzymatic hydrolysate was quantified by the glucose oxidase–peroxidase (GOPOD) method at 510 nm [19].

(4) $$RDS\left(\%\right)=\frac{\left(G_{20}-G_{F}\right)\times 0.9}{TS}$$

(5) $$SDS\left(\%\right)=\frac{\left(G_{120}-G_{20}\right)\times 0.9}{TS}$$

(6) $$RSV\left(\%\right)=\frac{\left[TS-\left(RDS+SDS\right)\right]}{TS}$$

Here, *G*_20_ and *G*_120_ represent the quantities of glucose liberated after 20 min and 120 min of digestion, respectively.

#### 2.8.2. LOS Digestive Kinetics

WMA and its complexes (200 mg) were dispersed in 15 mL of sodium acetate buffer (0.2 M, pH = 5.2). In this buffer, samples were digested using 10 mL of an enzyme mixture containing 290 U/mL α-amylase and 15 U/mL amyloglucosidase at 37 °C with shaking at 150 rpm. At 0, 5, 10, 20, 30, 40, 50, 60, 90, 120, 180, 360, and 540 min, 0.5 mL of the digestion supernatant was immediately mixed with 4.5 mL of absolute ethanol to inactivate the enzymes and terminate the reaction. The released glucose equivalents at each time point were then determined using the GOPOD method. The percentage of hydrolyzed starch (*C*) was calculated using Formula (7). The obtained *C* values were then substituted into Formula (8) for the initial digestive kinetics to determine the equilibrium concentration (*C*_∞_) and the rate constant (*k*). Subsequently, the logarithmic transformation yielded Formula (9), which was used for precise derivative fitting. During this fitting process, the equation was decomposed to further derive the second-order difference equation, in which the y-values were defined as y = ln[(*C*_2_ − *C*_1_)/(*t*_2_ − *t*_1_), (*C*_3_ − *C*_2_)/(*t*_3_ − *t*_2_), …], and the corresponding x-values were defined as x = (*t*_2_ + *t*_1_)/2, (*t*_3_ + *t*_2_)/2, etc. The result of second-order difference was provided as Formula (10). Ultimately, the LOS analysis yielded parameters including the initial and final reaction times (*t*_0_ and *t_f_*), the rate constants (*k*_1_–f), and the equilibrium concentrations (*C_i∞_*, i = 1, 2, 3, …, f) for each individual hydrolysis phase [15].

(7) $$Percentage\;of\;hydrolyzed\;starch\;\left(\%\right)=\frac{\Delta A(Samples)}{\Delta A(Glucose\;Standard)}\times 25\times \frac{100\%}{200\;mg}\times \frac{162}{180}$$

(8) $$C=C_{f\infty }\left(1-e^{-kt}\right),\;C_{f\infty }\leq 100\%$$

(9) $$ln\frac{dC_{t}}{dt}=-kt+ln(C_{f\infty }k),\;C_{\infty }\leq 100\%$$

(10) $$C_{t}=\left\{\begin{array}{c} {C_{1}+C}_{1\infty }\left(1-e^{-k_{1}t}\right),\;0\leq t\leq t_{1}\\ {C_{2}+C}_{2\infty }\left(1-e^{-k_{2}t}\right),\;t_{1}\leq t\leq t_{2}\\ .......\\ {C_{f}+C}_{f\infty }\left(1-e^{-k_{f}t}\right),\;t_{f-1}\leq t\leq t_{f} \end{array}\right.$$

Δ*A* represents the absorbance, and Δ*A*(*sample*)/Δ*A*(*glucose standard*) denotes the glucose-release equivalent. The value 25 corresponds to the dilution factor of the 0.5 mL enzymatic hydrolysate in the GOPOD method, and 162/180 represents the conversion coefficient from starch to glucose.

*AUC* represents the area under the curve. The hydrolysis index (*HI*) and the estimated glycemic index (*EGI*) were calculated as follows.

(11) $$AUC=C_{f\infty }\left(t_{f}-t_{o}\right)-\left(\frac{C_{f\infty }}{k}\right)\left[1-exp-k\left(t_{f}-t_{0}\right)\right]$$

(12) $$HI=\frac{AUC\;(sample)}{AUC\;(white\;bread)}$$

(13) EGI=39.71+(0.549×HI)

### 2.9. Analysis of Principal Component and Neural Networks

The key physicochemical, structural, and digestive parameters were initially normalized using the Min–Max scaling method. Principal component analysis (*p* < 0.05) was then applied using an optimal dimensionality reduction analysis pattern, and the 2D projection was selected because the cumulative explained variance of the first two components exceeded 95. A correlation matrix was used to obtain descriptive statistics, including eigenvalues, eigenvectors, and principal component scores, and a biplot combining a loading plot and a score plot was constructed. To further identify the parameters that most strongly influenced slow digestibility, an extremely significant correlation network matrix graph (*p* < 0.01) was generated. The adjacency matrix and the network structure were visualized based on the Fruchterman–Reingold layout. The analysis was performed within an r^2^ range from 0.99 to −0.99 [7].

### 2.10. Statistical Analysis

Data processing was conducted in SPSS Statistics (v22.0, IBM Corp., Armonk, NY, USA), including analysis of variance, calculation of means, standard deviations, and principal component analysis. Each experimental condition contained five independent replicates. After ANOVA, group differences were examined using Tukey’s HSD test. All datasets (*n* = 5 per group) met the assumption of normal distribution, as confirmed by the Shapiro–Wilk test (*p* = 0.10–0.96). Extremely significant correlation network modeling was carried out using OriginPro 2022 (OriginLab Corp., Northampton, MA, USA).

## 3. Results

The influence of self-assembly behavior of high-SI nano amylopectin ternary complexes in the muti-scale structure and slow digestion mechanism was discussed in detail.

### 3.1. Construction and Verification of High-SI Nano Endogenous Amylopectin Ternary Complexes

#### 3.1.1. SI Optimization of Amylopectin Complexes

**Figure 2:**
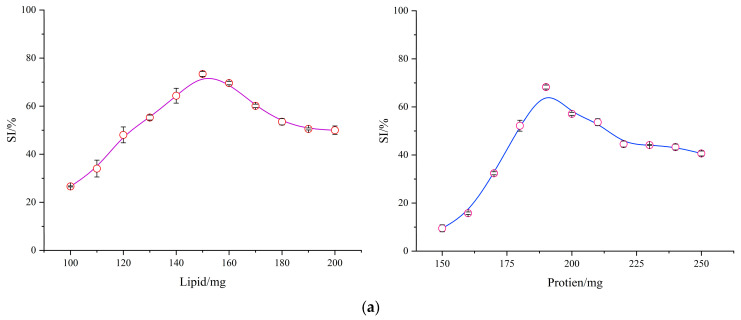

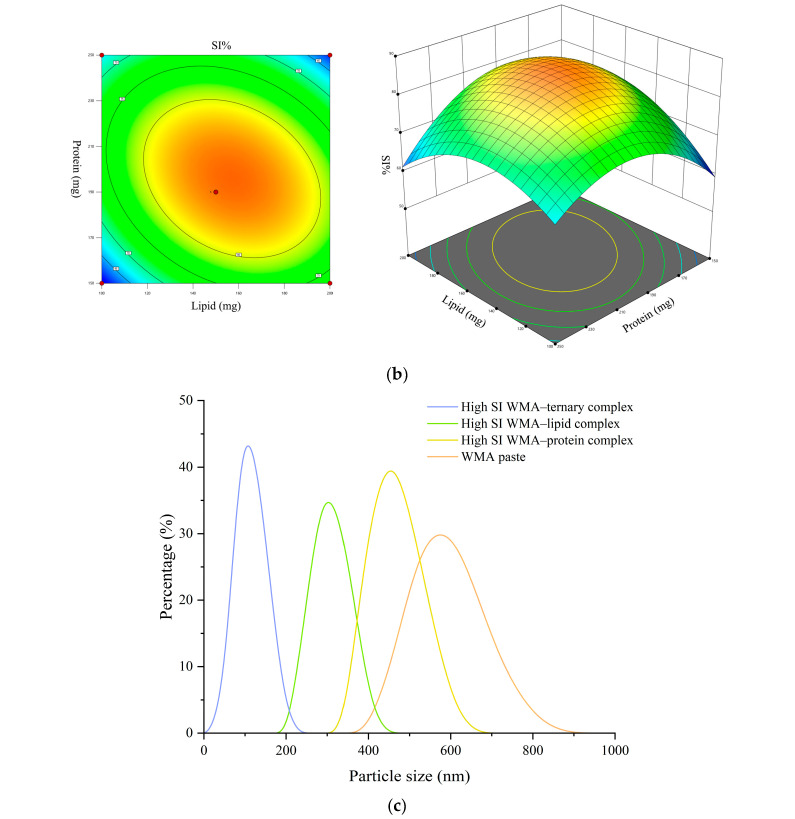
Construction of high-SI amylopectin complexes. (**a**) Single-factor results of amylopectin binary complexes. (**b**) Response surface experimental results of amylopectin ternary complexes. (**c**) Particle size distribution of the sample.

To obtain high-SI endogenous amylopectin ternary complexes through optimization, single-factor experiments were initially performed to determine the levels of ligand addition. The results were then subjected to automated nonlinear curve fitting, yielding the optimal SI values for WMA–lipid and WMA–protein complexes. As shown in Figure 2a, under fixed starch paste and water addition conditions, the optimal protein addition was 190 mg, yielding an SI of 68.25%, whereas the optimal lipid addition was 150 mg, with an SI of 73.40%. Furthermore, based on the single-factor parameters of the WMA–lipid and WMA–protein complexes, a response-surface analysis was designed to obtain the optimal SI of WMA ternary complexes. As illustrated in Figure 2b, under the same addition conditions of WMA paste and water, the optimal lipid and protein additions for the WMA ternary complex were 150 mg and 190 mg, respectively, producing an SI of 82.58%. The WMA ternary complex exhibited a markedly greater SI value than the WMA binary complexes (Figure 2a,b). This finding suggests that the extension of WMA side chains generated a deeper helical cavity, which facilitated the accommodation of additional endogenous lipid and protein residues during complex formation. The ligand residues refer to the fragments of WMA side chains, the hydrophobic alkyl chains and terminal carboxyl groups of lipids, and the polar side chains of proteins involved in the self-assembly process. The SI of endogenous WMA complexes was higher than that of the reported waxy starch–fatty acid complex (19.70–24.30%) [1]. This phenomenon might be attributed to the stronger synergistic effects among WMA, lipids, and proteins mediated by hydrogen bonding, free energy changes, electrostatic interactions, and van der Waals forces under optimal addition conditions and middle–high power ultrasonication assistance. These interactions reduced the glycosidic bond angles of branch chains, thereby decreasing branch-chain density and enlarging the molecular associations within the WMA complexes [7].

#### 3.1.2. Verification of High-SI Nano Endogenous Amylopectin Ternary Complexes

Figure 2c illustrates that the particle sizes of WMA paste and its binary complexes ranged from 285.67 to 588.12 nm, indicating that they were micro-nanoparticles. After undergoing amylopectin ternary self-assembly behavior, the particle size distribution of the high-SI WMA ternary complex markedly decreased to 99.87 nm, thereby confirming its nanoparticle characteristics. The significantly smaller particle size of the WMA ternary complex compared with other constituents may be attributed to the fact that during the self-assembly of WMA–lipid or WMA–protein complexes, only simple reactions are involved, in which the linear hydrophobic chains of lipids enter the helical cavities of amylopectin chains, or the hydrophobic amino acid segments of proteins adsorb onto the hydrophobic regions on the starch surface, forming binary complexes through the establishment of hydrogen-bond networks [8]. When proteins and lipids are both present during the WMA ternary self-assembly process, their hydroxyl, amide, and charged groups of the polar side chains can simultaneously form hydrogen-bonding networks with starch hydroxyl groups [21]. The interfacial regulatory effect of proteins enables the lipid chains to enter the starch cavity of a fine structure more fully and more deeply. Therefore, compared with WMA binary complexes, the WMA ternary self-assembly process can simultaneously induce the formation of multiple lipid–starch chain complexing structures. At the same time, the amide regions of proteins interact with these lipid–starch chain structures through higher electrostatic, hydrophobic, and hydrogen-bonding interactions, generating a more compact molecular architecture [22,23]. This tightly packed structure markedly reduces the spatial volume of the amorphous medium, which causes the lower Rg compared with the binary complexes [2]. Therefore, the particle size of the WMA ternary complex was significantly lower than that of the other constituents. The particle size distribution of high-SI nano WMA ternary complex was markedly lower than that of maize starch binary complexes and potato starch ternary complex (144–3774 nm) in the previous research [8,22]. This discrepancy may be explained by the fact that, during the self-assembly process, WMA exhibited a higher density of amylopectin branch clusters, a more orderly packing arrangement, and a reduced blocklet structure volume compared with the complexes reported in earlier studies. Although the high-purity nano WMA ternary complex has been successfully constructed and verified, the underlying mechanism governing its self-assembly behavior remains unclear. Accordingly, Zeta potential, DSC, and LOS kinetics analyses were utilized to test the key intermolecular forces driving self-assembly, encompassing electrostatic interactions, hydrogen bonds, van der Waals forces, and hydrophobic effects. And the characterization of macrostructural transitions and supramolecular reorganization during complex formation was detected by self-assembly characteristics and multi-scale structure analysis, while chemometrics were ultimately performed to elucidate the dynamic mechanism underlying the formation of the high-SI nano WMA ternary complex.

### 3.2. Self-Assembly Characteristics Analysis

**Figure 3:**
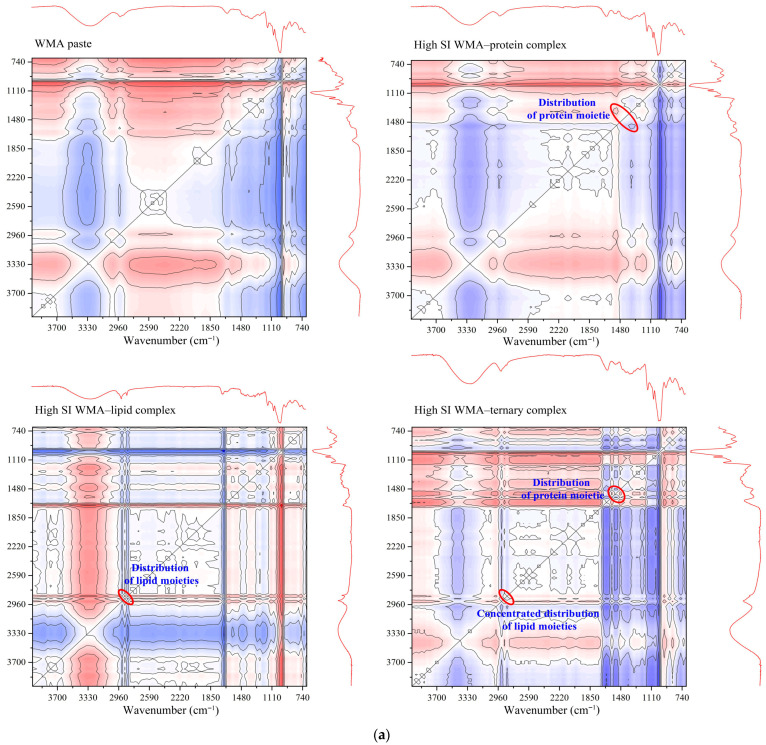

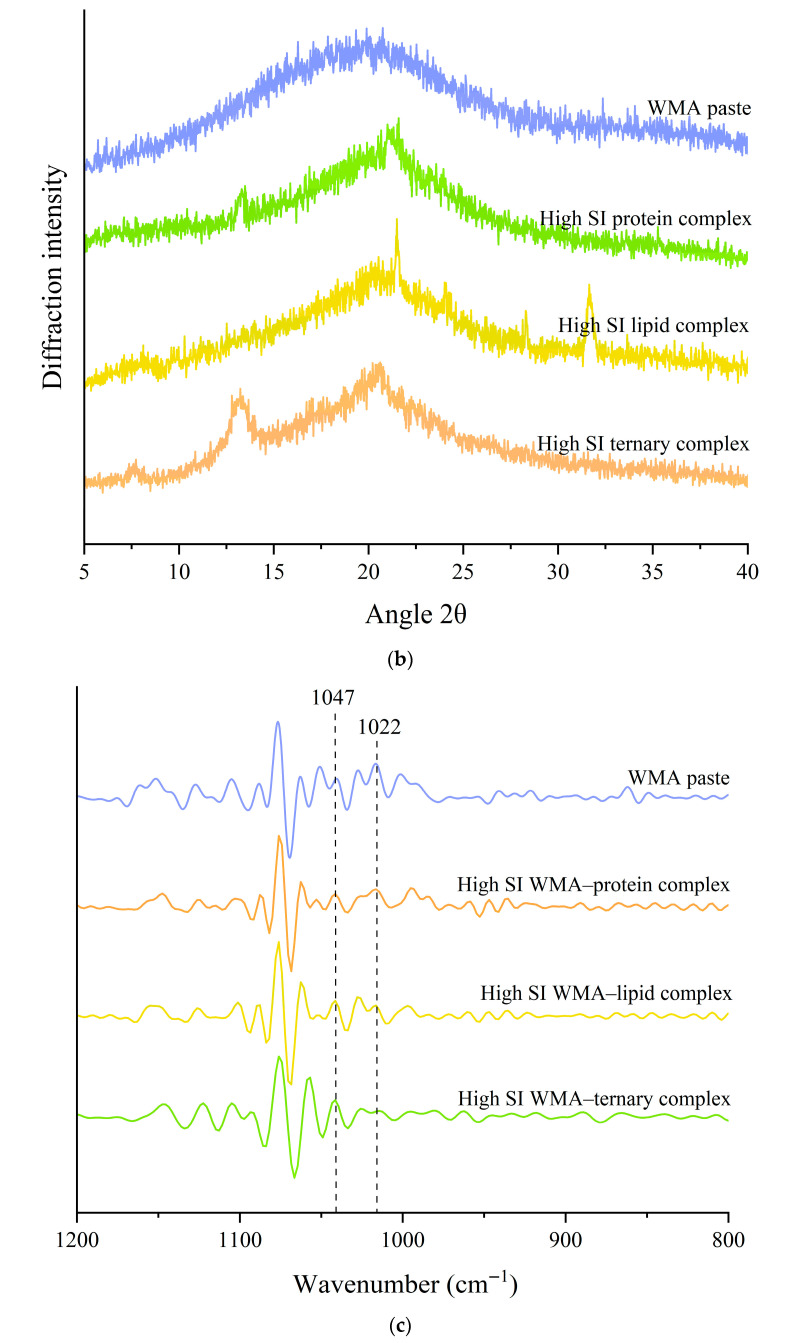

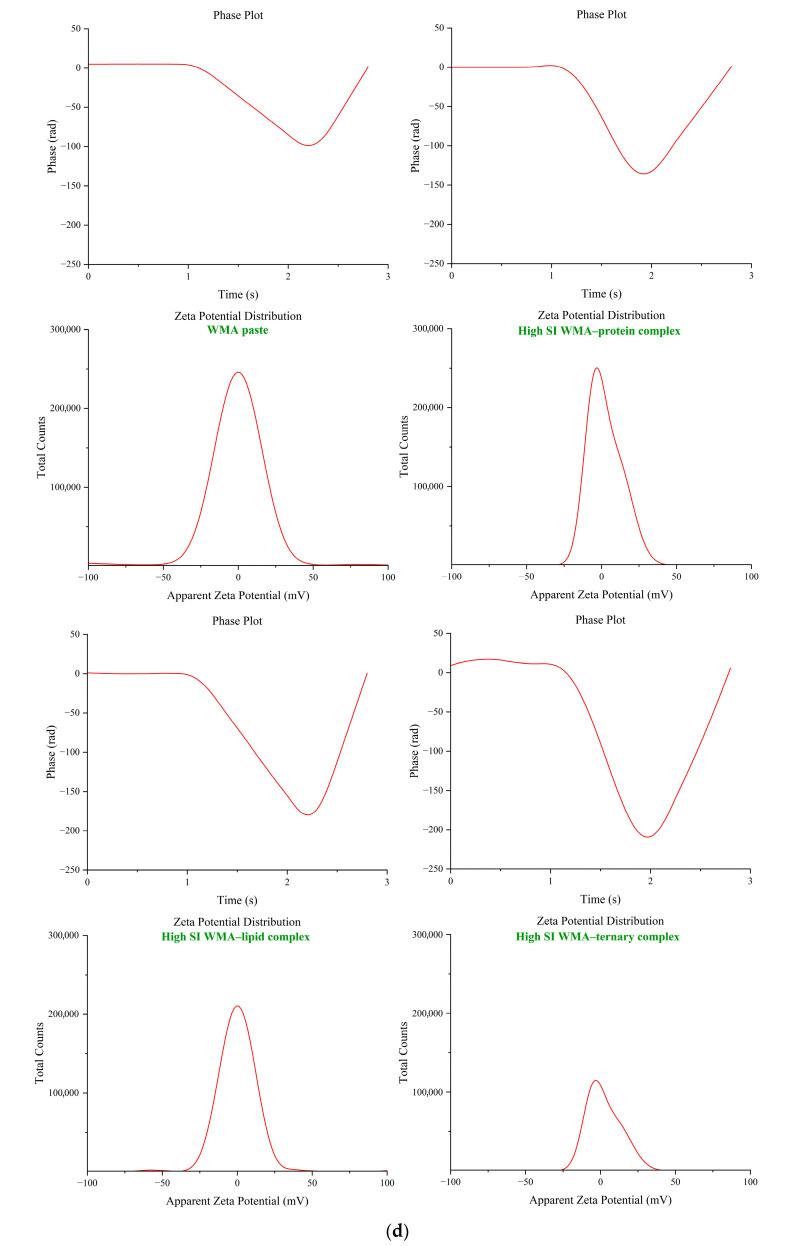
Self-assembly characteristics of WMA paste and the corresponding complexes: (**a**) 2D-COS total FITR spectrum; (**b**) XRD spectrum; (**c**) 800–1200 cm^−1^ FTIR spectra; (**d**) zeta potentiometric curve.

The self-assembly characteristics during self-assembly behavior were investigated using full-spectrum 2D-COS FTIR and XRD. As shown in Figure 3a, the 2D-COS FTIR analysis indicated that the WMA–lipid complex and WMA–protein complex displayed only a single characteristic absorption peak at 2846 cm^−1^ or 1540 cm^−1^, while WMA showed no absorption peak at these positions. The high-SI nano WMA ternary complex exhibited concentrated absorption peaks at 2846 cm^−1^ and dispersed characteristic peaks at 1540 cm^−1^, respectively (Figure 3a). These peaks corresponded to endogenous lipid alkyl groups within the internal starch cavity structure (2846 cm^−1^) and to amino acid residues and the amide II region of endogenous proteins either inside or outside the WMA cavity (1540 cm^−1^) [23]. These findings further demonstrated that interactions among the WMA cavity, dispersed amide regions, and concentrated alkyl groups progressively drove the transition of WMA paste toward the WMA ternary complex. Additionally, the XRD analysis revealed that WMA presented a typical amorphous broad diffraction pattern, accompanied only by a slight reflection near 20° (Figure 3b). This result indicated that the crystalline structure of the WMA paste was destroyed. After binary self-assembly behavior, the WMA–protein complex (2θ = 14° and 21°) and WMA–lipid complex (2θ = 21°, 28°, and 32°) exhibited V_a_ and a typical V crystal type, as described by Wang et al. [21]. These results were completely consistent with the report by Wang et al. [1] on wheat starch complexes. According to Dries et al. [24], after WMA cavity assembly with dispersed amide regions and concentrated alkyl groups, the crystalline diffraction was changed to V_h_-type (2θ = 7°, 14°, and 21°). It was verified that the packing patterns of WMA external side chains and the branching modes of internal side chains underwent significant rearrangements during ternary self-assembly.

The quantitative analysis of self-assembly characteristics during the assembly process was conducted using short-range FTIR, XRD, and Zeta potential. As shown in Figure 3b–d and Table 1, after the ternary self-assembly from WMA paste to the high-SI nano WMA ternary complex, the Zeta potential, short-range order, and Rc were significantly increased from −12.98 mV, 0.72, and 9.16% to −5.19 mV, 1.10, and 23.68%. The results could reveal that the WMA ternary self-assembly process increased both the depth and width of the WMA helical cavity, which facilitated the embedding of more ligand residues through increasing intermolecular electrostatic interactions, leading to higher SI and a more ordered folding of the spatial structure [21]. Therefore, WMA ternary self-assembly behavior formed spherulites more rapidly and in greater numbers and arranged more orderly in the amorphous medium. Moreover, the significantly higher zeta potential of the WMA ternary complex than that of the binary complexes might be due to its markedly higher self-assembly index (SI). This brought about that, compared to the WMA–lipid or WMA–protein binary complexes, a larger amount of WMA chains, lipids, and especially proteins participate cooperatively in the ternary assembly. The extensive participation of proteins introduces abundant polar side chains that contribute strong interfacial adsorption, thereby reshaping the local charge environment surrounding the carboxylated residues of WMA side chains as well as the hydrophobic alkyl chains and terminal carboxyl groups of lipids [25,26]. The Rc of the WMA ternary complex was markedly higher than that reported for corn starch complexes (12.56–19.80%) by Zhang et al. [13]. This discrepancy may be attributed to the formation of greater double-helix content and thicker crystalline lamellae in WMA complexes compared with those in corn starch complexes after self-assembly [15]. In addition, changes in the complexation characteristic parameters of starch and its complexes can lead to significant differences in supramolecular structure, physicochemical properties, SDS content, and LOS digestion kinetics. Therefore, the present study further investigated the multi-scale structure, thermodynamic properties, and in vitro digestibility of the samples.

### 3.3. Supramolecular Structure Analysis

#### 3.3.1. Helical Conformation Analysis

**Figure 4:**
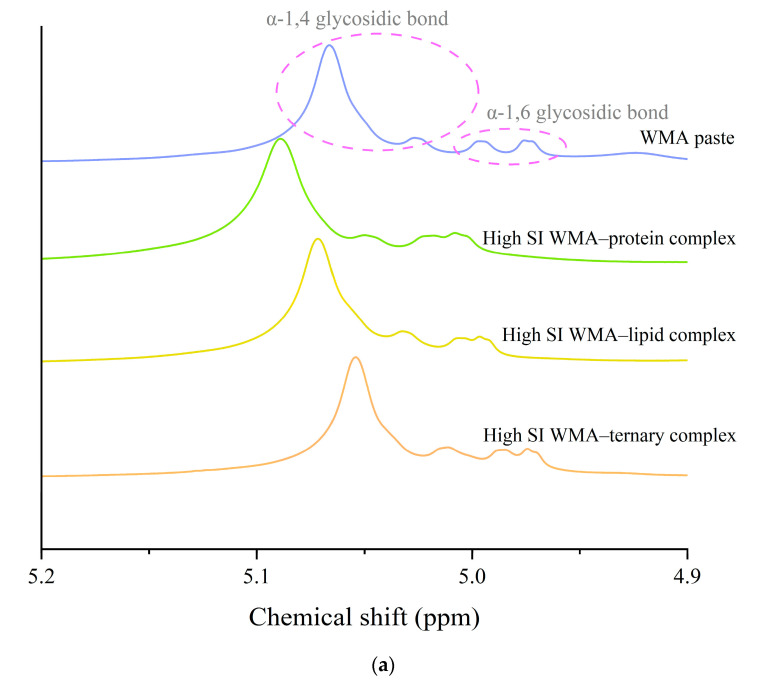

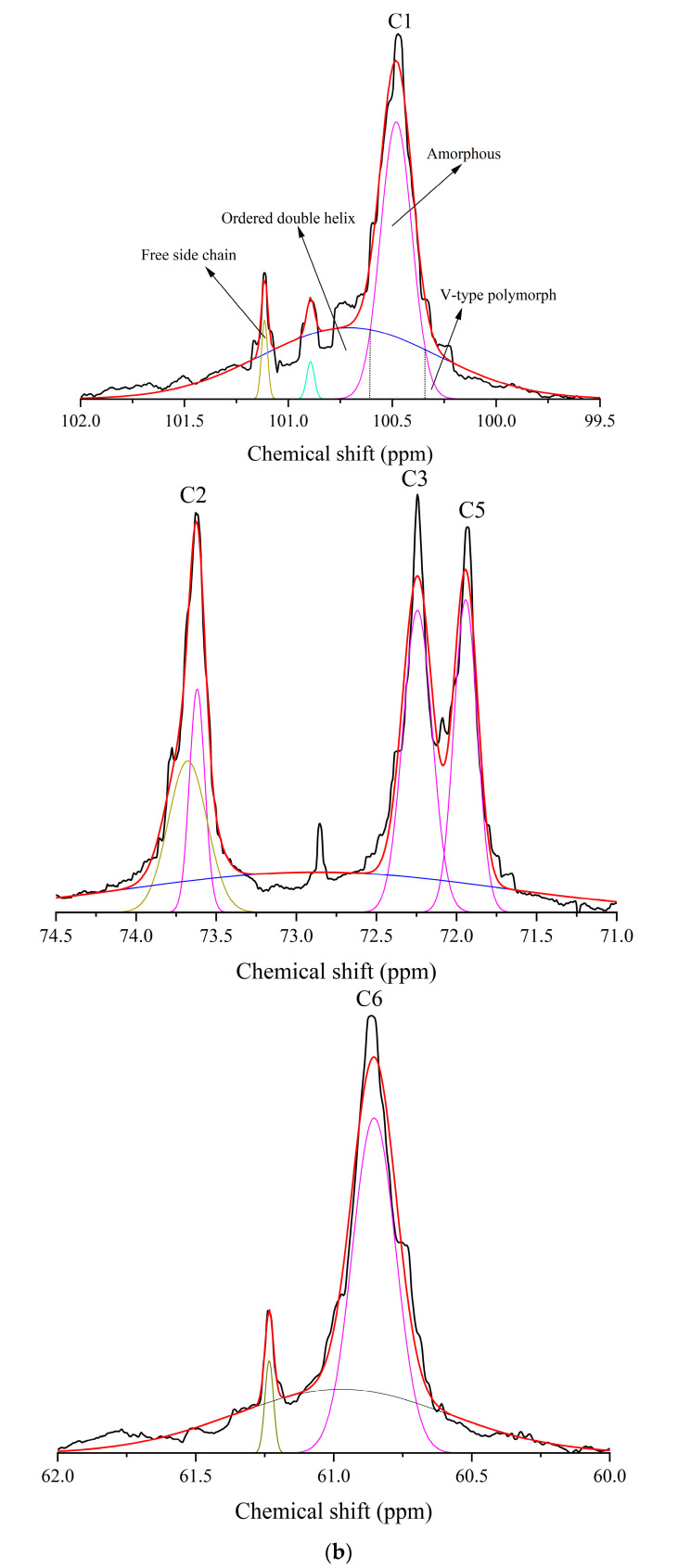

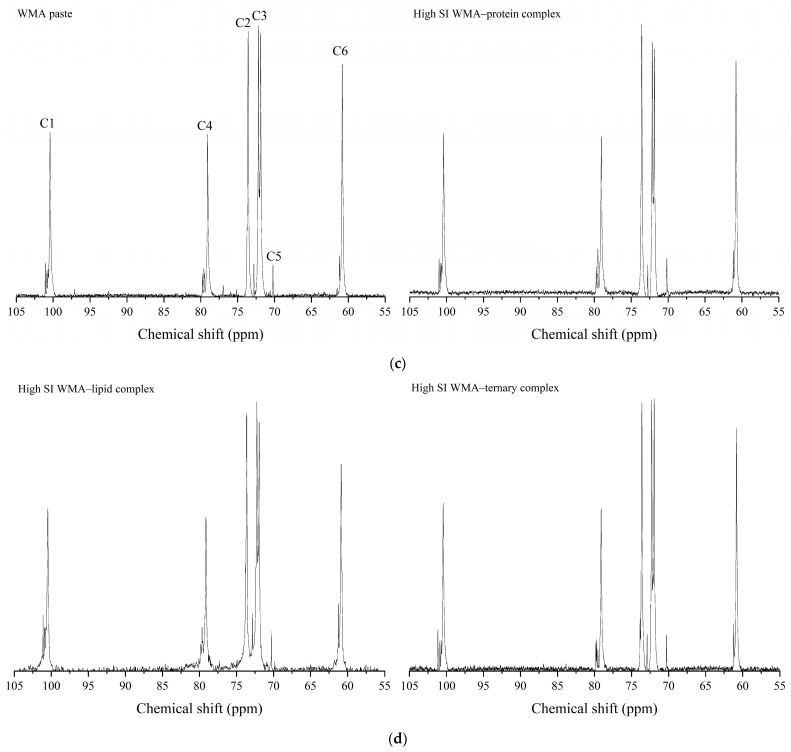
The helical conformation of WMA paste and the corresponding complexes: (**a**) 1H NMR spectra. (**b**) Deconvolution of the sub-spectrum into individual peaks of NMR ^13^C spectra. (**c**) ^13^C NMR spectra of WMA paste and WMA–protein complex. (**d**) ^13^C NMR spectra of WMA–lipid and WMA ternary complex.

Helical conformation changes induced by self-assembly were characterized by ^13^C and ^1^H NMR. As shown in Figure 4a–d and Table 2, after the transition of WMA paste into the high-SI nano WMA ternary complex through self-assembly, the relative contents of α-1,6 glycosidic bonds, CH_2_OH groups (free side chains across crystalline lamellae), double helices (Dhelix), and V-type polymorphs (Vp) increased from 5.35–10.09%, 5.20–11.15%, 19.55–33.55%, and 1.61–8.93% to 12.34%, 14.36%, 50.20%, and 11.14%, respectively. In contrast, the relative contents of α-1,4 glycosidic bonds and amorphous components (Ac) decreased from 89.91–94.65% and 57.52–78.84% to 87.66% and 38.66%, respectively (Figure 4a–c and Table 2). This phenomenon may be explained by the enhanced accessibility of the free (4→1)-α, (1→4)-α, (1→6)-α, and (6→1)-α–CH_2_O bridge groups of WMA chains to lipid alkyl chains and folded protein subunits during ternary self-assembly. As a result, protein and lipid ligands introduced polar residues and contributed to a higher Zeta potential, which induced hyperconjugation effects and catalyzed the conversion of glycosidic oxygen atoms of short chains in WMA toward the non-reducing 4-OH termini of long chains. Consequently, the long chains of WMA served as complexation sites to facilitate the high SI and the formation of numerous molecular helices (V6/7/8 II/I-type complexes), enhancing Rc and short-range order during ternary self-assembly. The α-1,6 glycosidic bond and Dhelix contents of high-SI nano WMA ternary complex were comparable to those of previous reports for breadfruit starch ternary complexes (10.05–13.02% and 34.15–37.06%) [7].

#### 3.3.2. Semicrystalline Lamellar Structure

**Figure 5:**
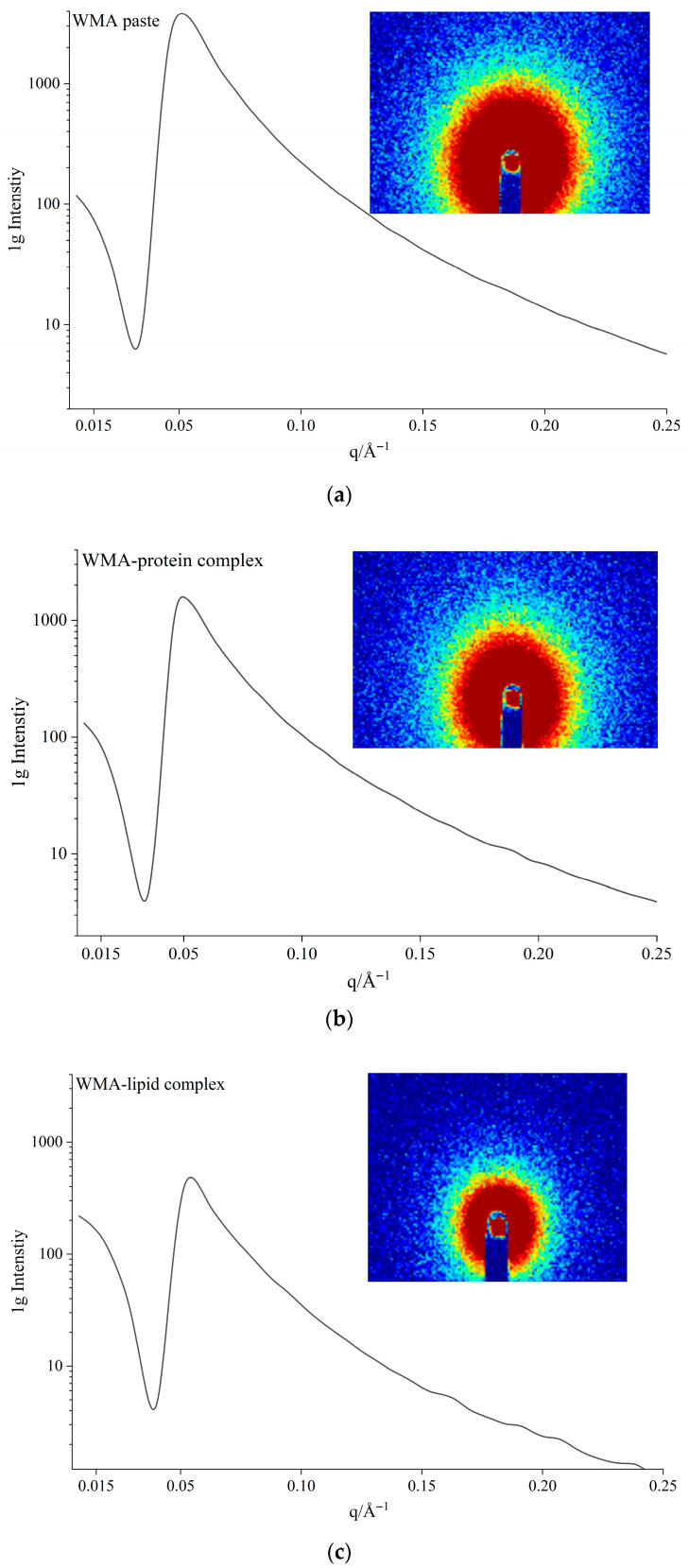

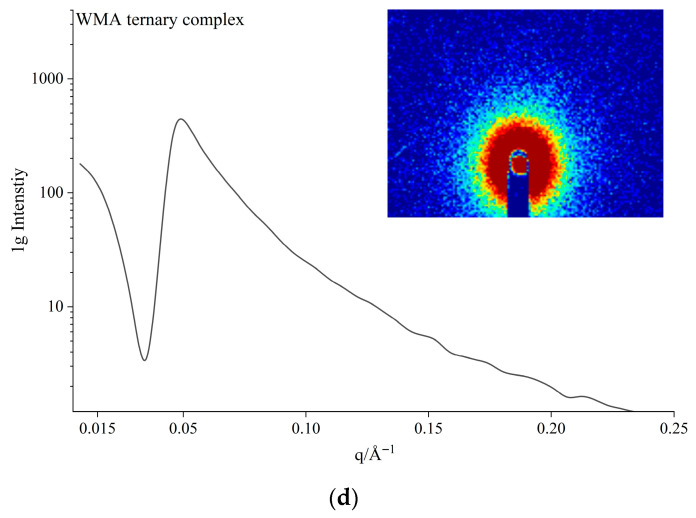
The 1D and 2D SAXS diffraction curves: (**a**) WMA paste; (**b**) WMA–protein complex; (**c**) WMA–lipid complex; (**d**) WMA ternary complex.

The semicrystalline lamellar structures of the samples could be analyzed using SAXS. As displayed in Figure 5a–d, the 1D curve of all samples displayed a broad peak at approximately 0.05 Å^−1^. This confirmed that an approximately 13 nm mass fractal structure existed in WMA paste and its WMA complexes [15]. The 2D figure shows an irregularly and inhomogeneously near-circular scattering ring (Figure 5), indicating an anisotropically non-periodic structure of WMA paste and its WMA complexes [2].

As shown in Table 3 and Figure 5a–d, during the self-assembly process from WMA paste to the high-SI nano WMA ternary complexes, decreases in *d* (10.88 to 3.97 nm), *d_a_* (5.55 to 1.98 nm), *d_c_* (5.33 to 1.99 nm), and *D_m_* (2.89 to 1.44) together with an increase in *α* (−2.89 to −1.44) were observed. According to previous reports [7,15], these results indicate that, during the formation of the high-SI nano WMA complex, the electron cloud density among the helices or the free amylopectin side chains progressively became highly concentrated, which contributed to an increase in SI and led to an increased density and reduced thickness of the crystalline lamella. At the same time, the van der Waals interactions among the inter-lamellar long side chains were strengthened, thereby reducing the volume of the amorphous lamellar and semicrystalline lamellar. In addition, the number of ordered helices increased, and the spatial arrangement of free side chains became more ordered, causing the microcrystalline lattice network inside the semicrystalline lamella to transform from a disordered to a more ordered state. Consequently, *D_m_* decreased while *α* increased after WMA ternary self-assembly. Previous reports found similar outcomes that the *d_a_* and *d_c_* of rice starch ternary complexes were 1.84–2.23 nm and 1.75–2.02 nm [15]. Lu et al. [17] also found the decreased *d_c_* (4.42 to 2.80−3.55 nm) of maize starch after complexing.

#### 3.3.3. Molecular Structure Analysis

**Figure 6:**
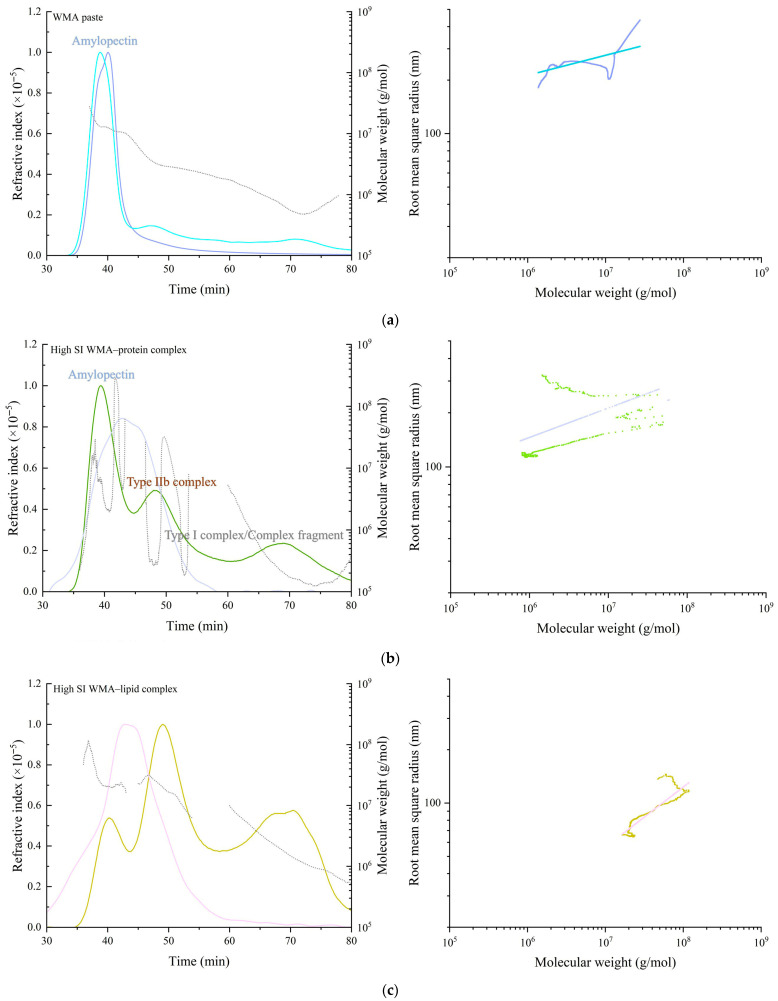

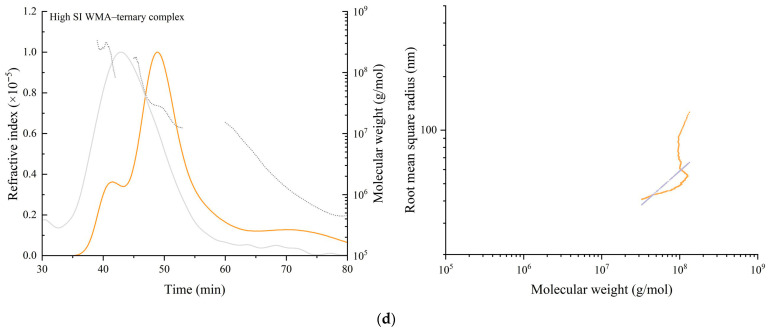
Molecular weight distribution (left) and molecular configuration spectrum (right) of WMA paste and the corresponding complexes. (**a**) WMA paste; (**b**) WMA–protein complex; (**c**) WMA–lipid complex; (**d**) WMA ternary complex.

The molecular configuration and conformation parameters of WMA paste, WMA binary complexes, and WMA ternary complex were characterized using HPSEC-RI-LS. As shown in Figure 6a–d, the RI chromatogram of WMA paste showed only a single main peak with a molecular weight ranging from 10^5^ to 10^8^ Da, suggesting a relatively homogeneous structure with high amylopectin purity and confirming that neither main chains nor branch chains were cleaved during gelatinization. Following ternary self-assembly, the RI of the high-SI nano WMA ternary complex revealed a transformation into a “multi-phase non-periodic structure” characterized by three distinct peaks corresponding to WMA paste (≈10^6^–10^9^ Da), type IIb WMA complexes (≈10^7^–10^9^ Da), and type I WMA ternary complexes or residual complex structures (≈10^6^–10^7^ Da). These effects resulted in the transformation of the irregular and fluffy spherical molecular conformation of WMA paste (*ν* = 0.18) into nearly spherical conformations (*ν* = 0.35–0.49) upon its transition into the high-SI nano WMA ternary complex (Figure 6 and Table 4) [2]. These transformations were primarily attributed to the displacement of microcrystalline nuclei within the multiphase periodic structures during self-assembly, which consequently altered the molecular spin orientation.

Upon the transition of WMA paste into the high-SI nano WMA ternary complex through self-assembly, quantitative analysis of configuration and conformation showed that Mn, Mw, *ν*, and *ρ* increases of 2.11–3.41 × 10^7^ Da, 3.78–4.74 × 10^7^ Da, 0.18–0.41, and 15.51–64.80 g mol^−1^ nm^−3^ to 4.07 × 10^7^ Da, 5.25 × 10^7^ Da, 0.49, and 125.34 g mol^−1^ nm^−3^, respectively (Figure 6a–d and Table 4). In contrast, the Rg and PI values decreased from 134.55 nm and 1.79 to 74.82 nm and 1.28. Based on previous reports [8,25], these transitions during WMA ternary self-assembly could be attributed to increases in SI and amylopectin branch density, which promoted a rise in both the number and compactness of ordered helices within the crystalline lamellae. As a result, a more ordered and compact microcrystalline network was formed, producing larger molecular spin angles and more uniform spin orientations. This process also reduced the dimensions of V-type polymorphic units and the thickness of crystalline lamellae. Consequently, the molecular weight, internal molecular density, and molecular uniformity increased after WMA ternary self-assembly. Zhang et al. [26] reported that the Rg and Mw of maize starch ternary complexes were 20–100 nm and 10^6^–10^7^ Da, respectively, which were considerably lower than those of high-SI nano WMA ternary complexes. Such differences may result from the higher degree of polymerization of amylopectin backbones and the greater spatial density of branch-chain clusters in WMA ternary complexes compared with other maize starch complexes.

#### 3.3.4. Characteristics of Microstructural Morphology and Nanoscale Surface Texture

**Figure 7:**
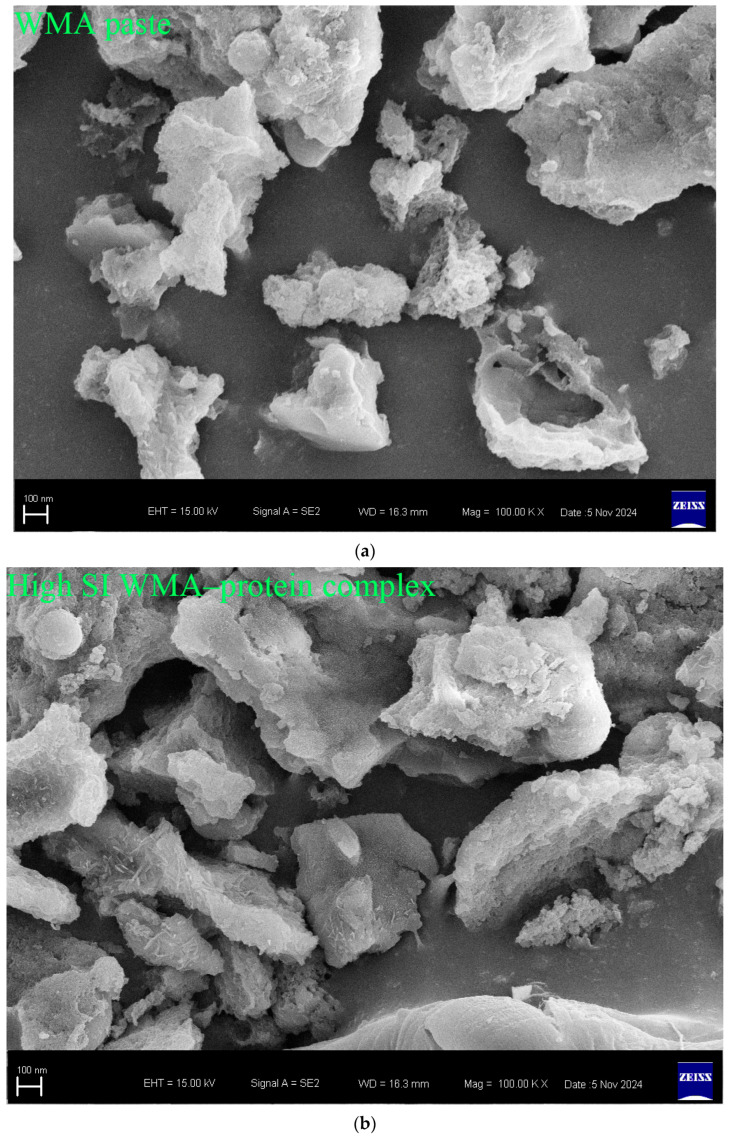

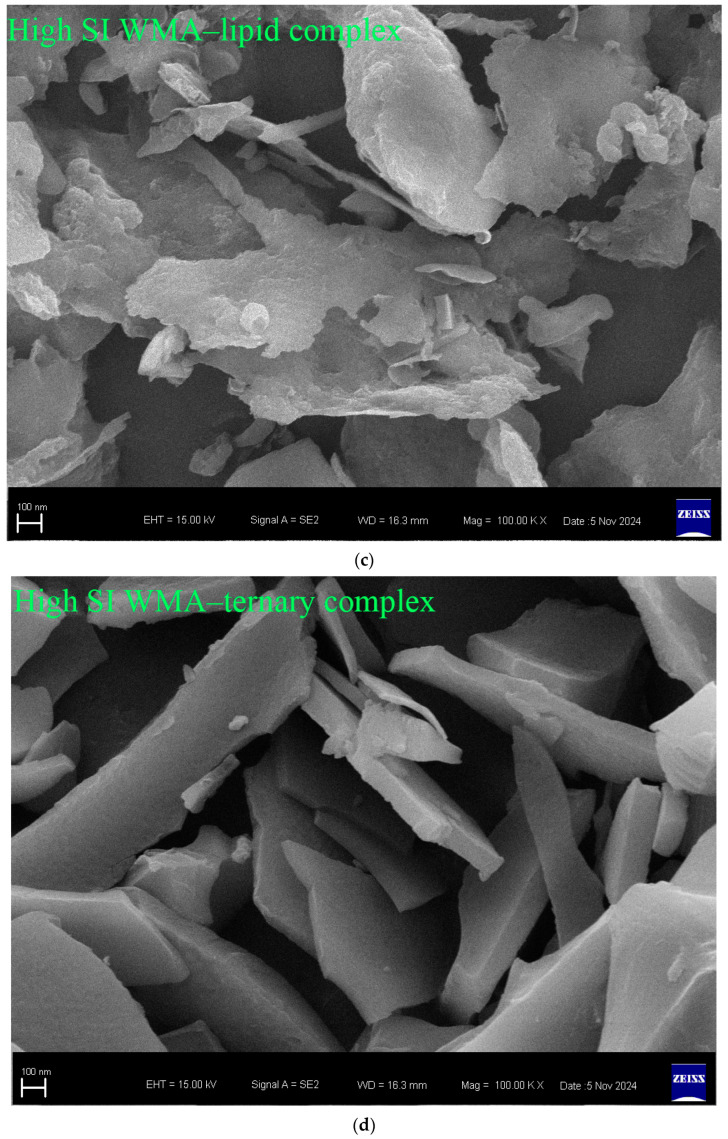
Particle morphology of various samples. (**a**) WMA paste; (**b**) WMA–protein complex; (**c**) WMA–lipid complex; (**d**) WMA ternary complex.

**Figure 8:**
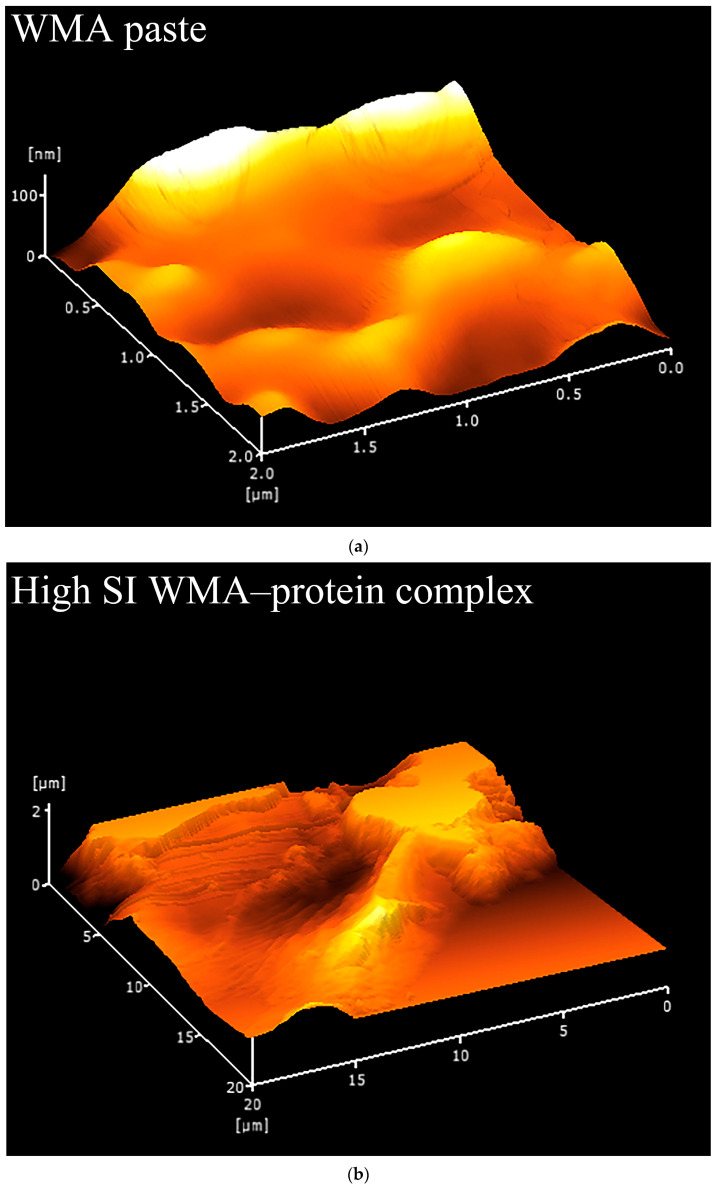

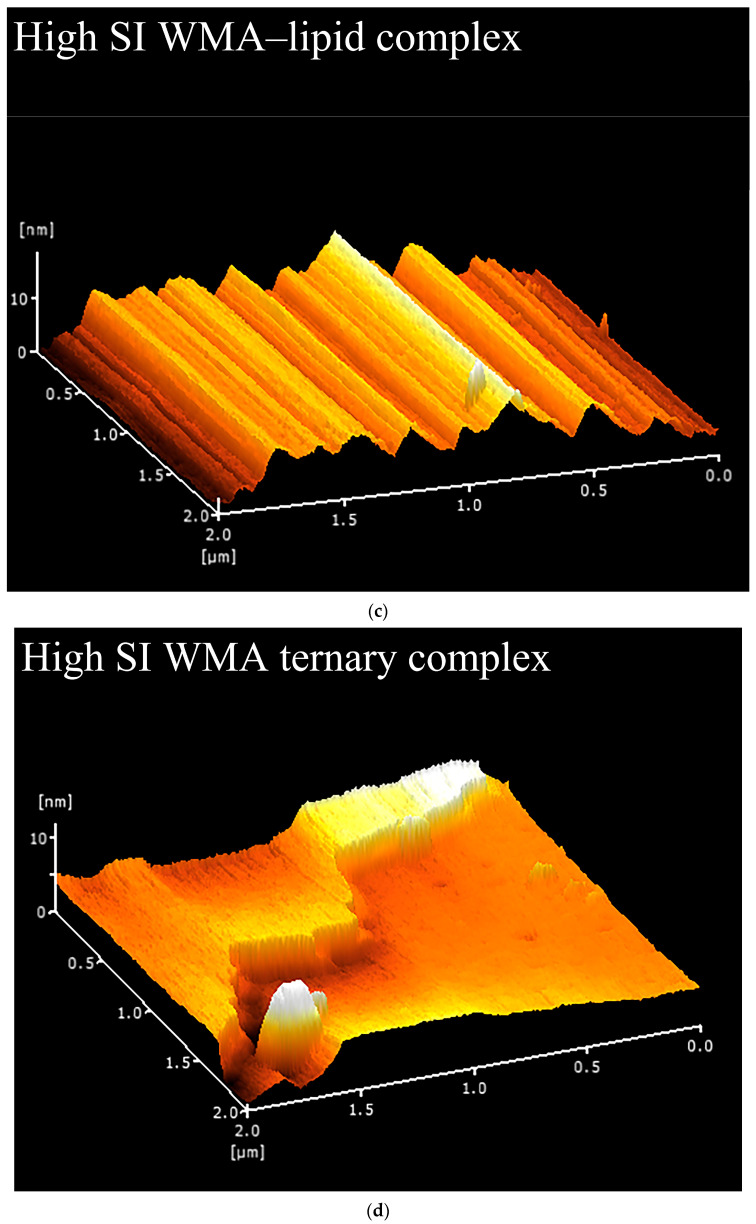
Nano surface characteristics analyzed by 3D surface scan. (**a**) WMA paste; (**b**) WMA–protein complex; (**c**) WMA–lipid complex; (**d**) WMA ternary complex.

Fewer surface pores and protrusions, reduced sponge-like features, lower surface fractal dimensions, and diminished overall roughness were observed in the SEM images after the high-SI nano WMA ternary self-assembly (Figure 7a–d). Meanwhile, the nanoscale emulsion-like protrusions and surface enzymatic channels observed by SEM on the surfaces of WMA paste and its complexes could be further analyzed by AFM. Firstly, as shown in the phase image and stiffness map in Figure 8a–d and Figure A1, the WMA ternary complex exhibited lower AFM scanning energy and entropy than the WMA paste and its binary complexes, indicating that a nanoscale surface with greater homogeneity and a lower fractal dimension was formed through ternary self-assembly [20]. These findings further validated the SEM observations described above. Furthermore, the 3D AFM images in Figure 8a–d and Figure A1 and Table 5 revealed that the Rq of the high-SI nano WMA ternary complex (8.01 nm) was significantly lower than that of the WMA paste and its binary complexes (9.22–17.64 nm), indicating that the size of the “blocklet” structures continuously decreased during ternary self-assembly. Moreover, as the self-assembly of the WMA ternary complex proceeded, the gradual increases in energy and homogeneity indicated enhanced particle uniformity and structural strength (Table 5 and Figure A1). The decreases in entropy, fractal dimension, and contrast during complexation were associated with a progressively smoother surface, higher compactness, and higher surface stability, accompanied by reduced surface roughness (Table 5 and Figure A1). These might be related to the loss of ordered WMA chain structures during the early stage of WMA self-assembly. In the later stage of self-assembly, the increased electron cloud density between O_6_ atoms and adjacent O_2_/O_3_ sites along the WMA chains strengthened the Zeta potential. Consequently, the substantial increase in complexing sites on side chains spanning the crystalline lamellar caused the rising compactness of amylopectin branch clusters. This strengthened the intra- and intermolecular non-covalent interactions and reduced the spatial distance between the amorphous and crystalline lamellae, thereby increasing the internal molecular density and the surface microstructure compactness of the particles [15,20]. Ultimately, the “blocklet” size was reduced, and textural features were refined after WMA ternary self-assembly. In this study, the Rq of the ternary complex was similar to that reported for breadfruit starch ternary complexes (5.36–7.92 nm) [7]. Furthermore, the blocklet structure also functions as a hydrolytic channel connecting the starch surface to its internal matrix, which is strongly correlated with thermodynamic behavior and in vitro enzymatic digestibility [20,21]. Therefore, the present study further examined gelatinization characteristics, SDS content, and digestion kinetics.

### 3.4. Gelatinization Characteristics

**Figure 9:**
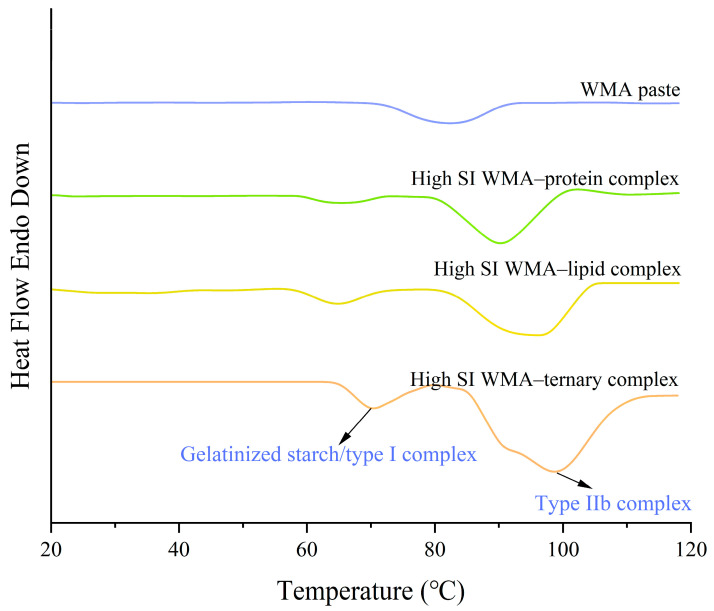
DSC curves of starch samples.

The gelatinization properties were investigated using DSC. The DSC analysis revealed a structural transition following the high-SI nano WMA ternary self-assembly, where the single microcrystalline melting peak of WMA paste evolved into two distinct melting peaks (Figure 9 and Table 6). In the high-SI nano WMA ternary complex, peak I and peak II corresponded to the semicrystalline melting peak of type I complexes or starch residue fragments and type IIb complexes, respectively, which was consistent with the “multi-phase non-periodic structure” characteristics observed in the molecular weight distribution results [2]. Among them, peak II represented the dominant peak of the complexes. Among them, peak II represented the dominant peak of the complexes. The To, Tp, Tc, R, and ΔHg of peak II increased from 70.06–81.86 °C, 81.84–95.17 °C, 92.18–106.18 °C, 22.12–24.32 °C, and 9.94–15.11 J g^−1^ to 84.57 °C, 98.65 °C, 114.37 °C, 29.80 °C, and 18.58 J g^−1^, respectively, as a result of WMA paste into the high-SI nano ternary complex (Figure 9 and Table 6). According to previous reports [15,20], the increase in gelatinization parameters observed in the WMA ternary self-assembly process could be attributed to the rising SI, hydrogen-bonding force, and van der Waals interactions. These factors promoted the formation of V6/7/8 helices, strengthened the ordering of helical arrangements, and improved the homogeneity of helical structures. As a result, the nanoparticle morphology developed progressively toward a compact and smooth surface microstructure. Chao et al. [8] reported that the Tp of potato starch ternary complexes was approximately 100 °C, similar to the results of the high-SI nano WMA ternary complex. However, the ΔHg obtained in this study was significantly higher than the reported values (4.3–10.5 J g^−1^). This difference might be attributed to the distinct water-holding capacity in the intramolecular hydroxyl group activity between WMA ternary complexes and potato starch ternary complexes.

### 3.5. Viscous Characteristics Analysis

**Figure 10:**
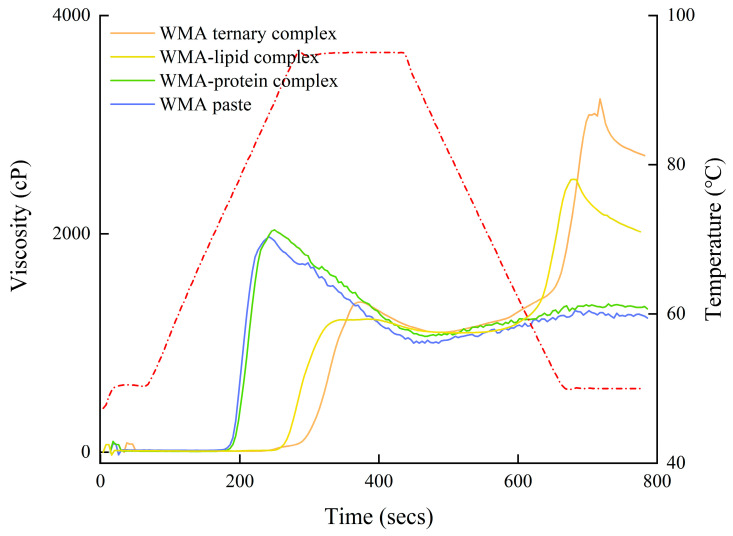
Viscous characteristics curves of WMA paste and its ternary complex (red dotted line is the temperature–time profile recorded during the RVA analysis).

The outcomes of viscous characteristics were analyzed to investigate the gel-network properties of the samples. As shown in Figure 10 and Table 7, the trough viscosity (TV), setback viscosity (SBV), final viscosity (FV), and pasting temperature (PT) of the high-SI nano WMA ternary complex (1442 cP, 1990 cP, 3432 cP, and 94.16 °C) were significantly higher than those of the other systems (1000–1098 cP, 229–1574 cP, 1229–2672 cP, and 73.5 °C). These results suggest that, during the WMA self-assembly process, increasing numbers of aliphatic hydrophobic tails and protein polar residues entered the WMA inter-helical voids, thereby strengthening SI, gradually increasing molecular-chain rigidity, and consequently improving the re-association ability of the WMA side chain during retrogradation [1]. This phenomenon enhanced the isotropic rearrangement capacity of disordered helices, increased double-helix stability, and accelerated the reorganization of the multi-scale molecular structure, ultimately resulting in higher SBV and PT [2,21]. Moreover, such changes increased Rc, elevated granular hardness, and improved shear resistance, thereby contributing to the increased TV and FV after high-SI nano WMA ternary complex [7]. Furthermore, the PT of all samples was lower than that of Tp; this might be due to the different desorption rate of the structure within the crystalline between DSC and RVA measurements. The TV, FV, and SBV of samples in the present research were broadly similar to previous reports for wheat starch complexes (684.0–1387.0 cP, 1464.0–2499.0 cP, and 188.0–1412.0 cP) [1]. In addition, the chaotic trend of peak viscosity (PV) and breakdown viscosity (BDV) was illustrated for WMA paste, WMA–protein complex, WMA–lipid complex, and high-SI nano WMA ternary complex (Figure 10 and Table 7). In addition, the chaotic trends in peak viscosity (PV) and breakdown viscosity (BDV) observed for WMA paste, the WMA–protein complex, the WMA–lipid complex, and the high-SI nano WMA ternary complex (Table 7 and Figure 10) may be attributed to the stochastic participation of varying numbers of V6/7/8-type helices in crystalline assembly during the self-assembly, which induces random fluctuations in molecular-chain flexibility and results in unstable granular swelling capacity under thermal mechanical conditions.

**Table 7:** The viscous characteristics of WMA paste and its ternary complex particles.

Samples	PV (cP)	TV (cP)	BDV (cP)	FV (cP)	SBV (cP)	PT (°C)
WMA paste	1970 ± 2.84 ^a^	1000 ± 5.52 ^d^	972 ± 3.17 ^a^	1229 ± 2.12 ^d^	229 ± 8.59 ^d^	73.5 ± 2.29 ^d^
High-SI WMA–protein complex	1219 ± 10.66 ^d^	1093 ± 3.61 ^bc^	126 ± 4.27 ^d^	2019 ± 12.30 ^c^	926 ± 8.11 ^c^	90.5 ± 0.96 ^bc^
High-SI WMA–lipid complex	1375 ± 9.58 ^c^	1098 ± 1.99 ^b^	277 ± 1.65 ^c^	2672 ± 6.62 ^b^	1574 ± 5.55 ^b^	91.25 ± 1.02 ^b^
High-SI WMA–ternary complex	1934 ± 3.81 ^ab^	1442 ± 10.66 ^a^	492 ± 5.52 ^b^	3432 ± 5.45 ^a^	1990 ± 1.68 ^a^	94.16 ± 1.10 ^a^

PV, Peak viscosity; TV, trough viscosity; BDV, breakdown viscosity; FV, final viscosity; SBV, setback viscosity; PT, pasting temperature. Different superscript letters within a column indicate statistically significant differences among the samples (*p* < 0.05).

### 3.6. Investigations of Slow Digestibility Characterization and LOS Kinetics

#### 3.6.1. Slow Digestibility Characterization

The slow digestibility characteristics during WMA ternary self-assembly were evaluated based on the analysis of in vitro digestion fractions. As shown in Table 8, the SDS and RS contents (19.86–28.05% and 7.49–28.82%, respectively) markedly increased to 43.28% and 32.24%, whereas the RDS content exhibited the opposite trend following ternary self-assembly. These results indicate that a large portion of RDS was converted into SDS, and a smaller fraction was converted into RS as the WMA paste underwent ternary self-assembly to form the high-SI nano complex. Consequently, the nanoscale WMA ternary complex can be classified as a high-SDS-content starch, suggesting its potential to moderate postprandial blood glucose elevation while allowing a sustained release of nutrients to meet essential physiological demands. According to previous reports [15], RDS structures are mainly located in amorphous regions, whereas the SDS and RS structures are distributed within perfect and imperfect crystalline regions, which are predominantly governed by hydrophobic interactions and free energy. Therefore, the increases in SDS and RS contents during WMA ternary self-assembly could be attributed to the gradual increase in complexation sites, as well as the enhanced molecular re-association ability and density of branched side chains [4,5]. These changes raised steric hindrance and strengthened the hydrophobic interactions among WMA long chains, the amide I/II regions of proteins, and the carboxyl chains of lipids. This process markedly increased the compactness of the molecular gel network and reduced the enzymatic cleavage sites located at side chains and branching points, promoting the formation of enzyme-resistant perfect crystalline structures [2]. Consequently, after ternary self-assembly, the higher SBV was observed, and granule morphology became more compact and smoother, with a reduced surface enzymatic channel size. Moreover, the SDS content of high-SI nano WMA ternary complex was markedly higher than that previously reported for ternary complexes of corn starch, maize amylopectin, cassava starch, potato starch, and rice starch (9.39–32.94%) [15,27]. This difference may result from variations in the self-assembly sites and multi-scale structural formation mechanisms during the ternary self-assembly process.

#### 3.6.2. In Vitro LOS Digestive Kinetics and EGI Analysis

**Figure 11:**
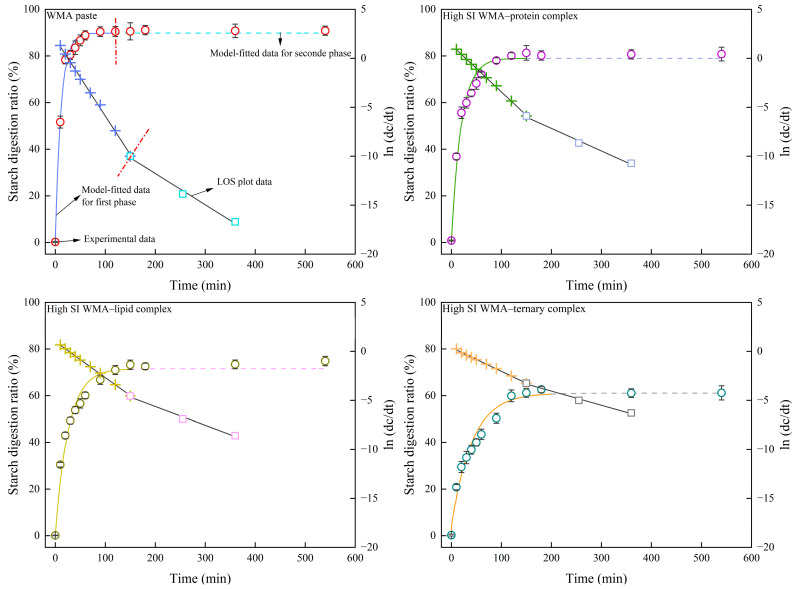
The LOS digestive kinetics curves of amylopectin paste and its complex samples. In the fitted kinetic curves, colored solid and dashed lines denote different digestion phases, and the LOS data points use the same color-phase correspondence.

Since the SDS content can markedly influence the dynamic digestion process, the LOS digestion kinetics of starch samples were further investigated. As shown in Table A1 and Table 9, compared to the LOS kinetics results, the *C*_∞_, HI, and EGI values of the classical kinetic approach showed no significant difference, whereas the *k* value was close to the mean of *k*_1_ and *k*_2_. This is attributed to the fact that the LOS kinetic fitting procedure requires an initial classical digestion kinetic fitting step, followed by derivative fitting and then second-order differencing. As a result, LOS produces multiple linear fitting segments. Therefore, compared with the classical digestive kinetics, the LOS kinetics allow a more rigorous and more accurate calculation of multi-stage *k* values and *C*_∞_ values from the slopes and intercepts, thereby yielding a more precise EGI. And for each sample, the LOS model was fitted to the in vitro digestion curves obtained from independent replicates, and the kinetic parameters *C*_∞_ and *k* were expressed as mean values with 95% confidence intervals calculated from replicate variability. As shown in Figure 11, all samples exhibited two second-order split fitting lines, indicating that the enzymatic hydrolysis process proceeded in two distinct stages. This suggests that the transformation from WMA paste to the high-SI nano WMA ternary complex did not alter the enzymatic hydrolysis pathway but substantially reshaped the original enzymatic cavity structures. These observations were consistent with DSC and molecular weight distribution results, which confirmed that the WMA ternary complex possessed a “multi-phase non-periodic structure.” However, although WMA paste exhibited multi-stage digestibility, it did not display “multi-phase non-periodic structure,” possibly due to the large variations in amylopectin chain length, branched degree, and branching pattern within its internal structure.

The two-stage kinetic parameters of the LOS model (*C_1∞_, C_2∞_, k_1_, k_2_, t_1_*, and *t_2_*) were obtained. The results revealed that, upon ternary self-assembly of WMA paste into the high-SI nano WMA ternary complex, RDS was converted into SDS and RS. Consequently, the values of *C_1∞_*, *C_2∞_*, *k*_1_, *k*_2_, HI, and EGI (70.95–89.39%, 71.54–90.13%, 3.68–7.89 × 10^−2^ min^−1^, 1.92–3.20 × 10^−2^ min^−1^, 83.99–105.30, and 85.82–97.52, respectively) markedly decreased to 60.21%, 61.06%, 2.43 × 10^−2^ min^−1^, 1.44 × 10^−2^ min^−1^, 71.83, and 79.15, whereas the *t_1_* showed an opposite trend (Figure 11 and Table 9). These results indicated that the glycemic-release equivalent decreased and the release rate gradually became steady after WMA ternary self-assembly, which can markedly reduce the risk of glycemic dysregulation. These phenomena could be attributed to the progressive increase in SI during the formation of the WMA ternary complex, which enhanced the toughness and rigidity of the trans-lamellar WMA chains and strengthened hydrophobic interactions between chains [3,27]. Then, a large number of enzymatic cleavage sites were gradually encapsulated, forming a highly cross-linked multi-scale molecular network and causing a substantial increase in FV and TV [28]. These features effectively delayed enzymatic penetration into glycosidic bonds, leading to the formation of abundant SDS structures. The *k*_1_ and *k*_2_ values of high-SI nano WMA ternary complex were significantly lower than those reported for rice starch ternary complexes (6.75–6.98 × 10^−2^ min^−1^ and 1.68–1.88 × 10^−2^ min^−1^, respectively) by Zhen et al. [15]. Moreover, the *C_2∞_* value of high-SI nano WMA ternary complex was markedly lower than that reported for kidney bean starch ternary complexes (80.44–92.12%) by Wu et al. [28]. These differences may be attributed to the higher density of semicrystalline lamellae and Rc in the nanoscale WMA ternary complex compared with rice and kidney bean starch complexes.

### 3.7. Relationship Between Slow Digestibility and Physicochemical Structural Characteristics

**Figure 12:**
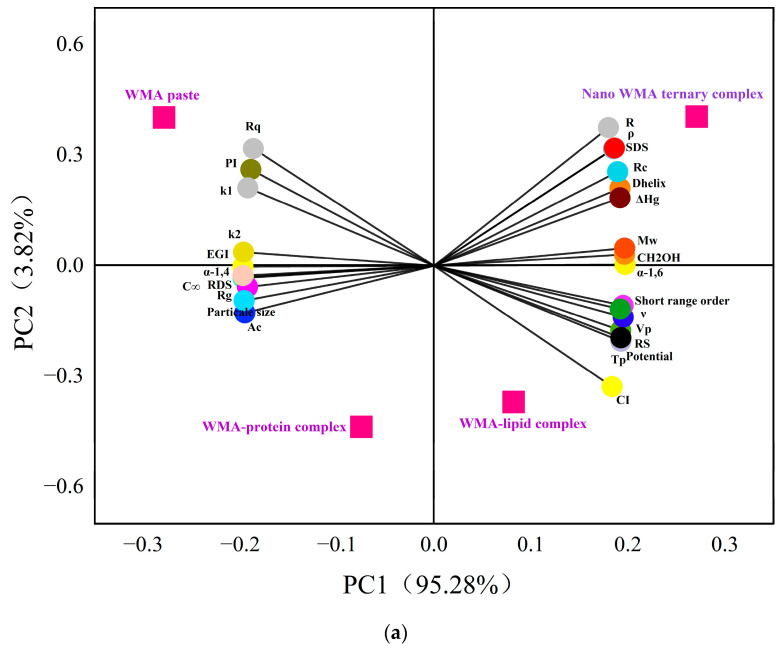

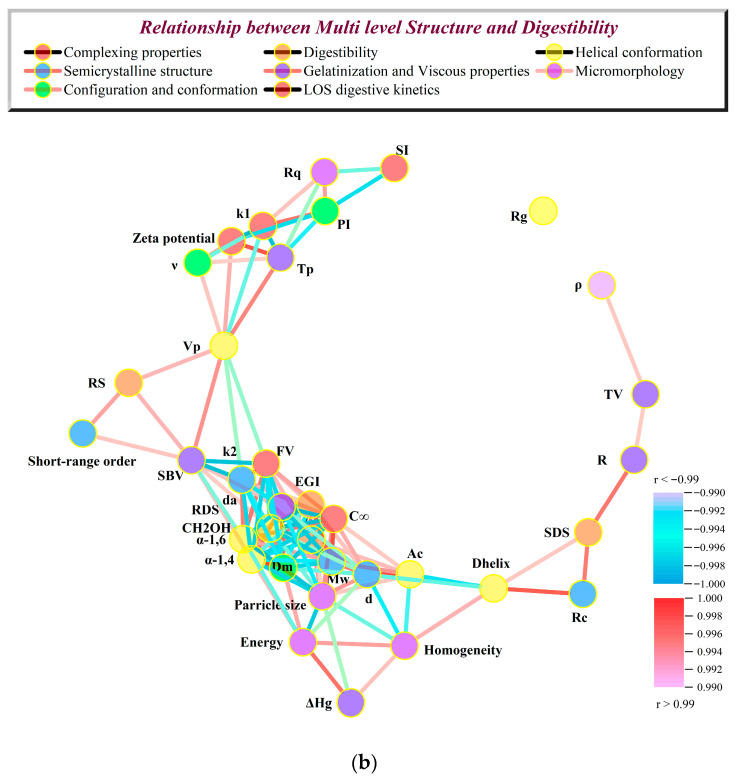
Relationship between in vitro digestive properties and multi-scale structures. (**a**) The analysis of 2D PCA; (**b**) extremely significant correlation network analysis.

Initially, 2D PCA was employed to reveal the relationships between slow digestibility and physicochemical structural characteristics during the formation of high-SI nano WMA ternary complex. Two-dimensional PCA demonstrated that WMA paste and its binary and ternary complexes were clearly separated in the 2D space, reflecting significant variations in in vitro digestibility and multi-scale structural characteristics induced by ternary self-assembly (Figure 12a). The cumulative contribution of PC1 and PC2 exceeded 99%, confirming that the 2D PCA model provided a comprehensive representation of the overall data structure (Figure 12a) [7]. The PCA plot further revealed strong positive correlations (angle < 45°, r > 0.80, *p* < 0.05) among Rq, PI, *k*_1_, *k*_2_, EGI, α-1,4 glycosidic bonds, *C*_∞_, RDS, Rg, particle size, Ac, *d*, *da*, and *D_m_*. Strong positive correlations (angle < 45°, r > 0.80, *p* < 0.05) were also observed among R, *ρ*, SDS, Rc, Dhelix, ΔHg, Mw, α-1,6 glycosidic bonds, CH_2_OH, short-range order, *ν,* Vp, RS, Tp, Zeta potential, SI, homogeneity, energy, TV, FV, and SBV. And Rq, PI, *k*_1_, *k*_2_, EGI, α-1,4, *C*_∞_, RDS, Rg, particle size Ac, *d*, *da*, and *D_m_* exhibited strong negative correlations with R, *ρ*, SDS, Rc, Dhelix, ΔHg, Mw, CH_2_OH, α-1,6, short-range order, ν, Vp, RS, Tp, zeta potential, SI, homogeneity, entropy, TV, FV, and SBV (angle > 45°, r < –0.80, *p* < 0.05) (Figure 12a). These results revealed that all parameters, including gelatinization characteristics, viscous characteristics, multi-scale structural features, and LOS kinetics, might significantly influence the formation of SDS during the formation of the high-SI nano WMA ternary complex.

Furthermore, an extremely significant correlation network analysis based on the Fruchterman–Reingold algorithm further showed that the adjacency matrix of the correlation network indicated two parameter regions (Figure 12b). Region I represented the high-clustering parameter zone, including Rq, PI, *k*_1_, *k*_2_, EGI, α-1,6, *C*_∞_, RDS, Rg, particle size, R, *ρ*, SDS, Rc, Dhelix, ΔHg, Mw, CH_2_OH, α-1,4, short-range order, *ν*, Vp, RS, Tp, zeta potential, and SI (Figure 12b). This indicated that most multilevel supramolecular structures and digestion kinetics parameters were extremely significantly correlated with SDS after WMA ternary self-assembly behavior (r > 0.99 or r < −0.99, *p* < 0.01) (Figure 12b). The results of Region I were consistent with the PCA results. Region II was composed of scattered peripheral points, including Rg, suggesting that Rg played an auxiliary role in influencing SDS. However, PCA indicated that Rg was significantly correlated with Region I (*p* < 0.05), whereas correlation network analysis did not show extremely significant correlations between Rg and Region I. This discrepancy may be attributed to the “multi-phase non-periodic structure” of the complexes, in which amorphous contents with varying sizes were randomly distributed within semicrystalline structures. Similarly, Zhang et al. [13] reported that in maize starch complexes, Rc increased from 12.56% to 19.80%, while ΔH increased from 3.21 to 9.88 J g^−1^. Chen et al. [29] reported that after lotus seed starch self-assembly, Tp increased from 81.5 °C to 123.9 °C, while *k* decreased from 7.1 × 10^−2^ min^−1^ to 1.9 × 10^−2^ min^−1^, accompanied by an increase in SDS content from 14.1% to 40.1%. Overall, these results indicated that during the high-SI nano WMA ternary self-assembly process, the amylopectin side chains exhibited progressively possibly enhanced binding affinity and interaction energy with lipid and protein residues. It could be inferred that the strengthened molecular interactions promoted the formation of ordered helical conformations, which further developed into tightly packed type I/IIb crystalline spherulites embedded within the amorphous. This suggested that hierarchical reorganization markedly improved the degree of molecular order, leading to reductions in the lamellar thickness and resulting in nano morphologies characterized by denser and smoother surfaces. Simultaneously, the enhanced structural compactness, increased thermal stability, and raised gel-network rigidity perhaps led to the increasing SDS content after ternary self-assembly behavior, thereby significantly suppressing the enzymatic hydrolysis rate and slowing the glucose release.

### 3.8. General Discussion

In summary, comprehensive analyses and discussions were conducted through 2D PCA, and a correlation network of high-SI nano WMA ternary self-assembly behavior could be used to verify the findings. These integrated approaches aimed to derive the most precise conclusion regarding the WMA ternary self-assembly behavior in the slow digestion mechanism. Initially, the molecular weight distribution and 2D-COS FTIR spectra exhibited distinct self-assembly characteristic peaks in the high-SI nano WMA ternary complex, confirming the successful formation of the WMA ternary complex. Furthermore, the content of α-1,6 glycosidic bonds showed a significant increase during WMA ternary self-assembly, suggesting that under ultrasound-assisted and alkaline self-assembly conditions, the hyperconjugation effects of polar residues intensified the grafting of α-1,4 glucan residues onto the non-reducing ends of α-1,6 glycosidic bonds or backbone [21]. And more O_2_–O_6_ positions within the α-1,4 glycosidic bonds and CH_2_OH groups on WMA chains acted as self-assembly sites that interact with lipids, ionizable carboxyl groups, and proteins’ amide region through non-covalent interactions [7]. This perhaps led to an increase in SI and Zeta potential, as well as the formation of orderly arranged long or short Dhelix structures within the WMA ternary self-assembly process. Such structural evolution generated type I and type II complexes and produced new peaks in the molecular weight distribution, indicating the formation of a “multi-phase non-periodic system” [15]. It also promoted isothermal crystallization and facilitated the formation of a large number of compact Vp structures [30]. Consequently, the crystallinity of WMA gradually transformed from broad diffuse scattering to ordered and compact V_h_-type crystallinity, accompanied by a conformational transition from an irregular and fluffy spherical to spherical conformations [31,32]. Therefore, the decreased *d* and *d_a_* were shown due to the strengthened conjugation reactions (inter-chain coupling) and the increased electron cloud density within semicrystalline lamellar. Such changes in the semicrystalline structure exhibited decreased PI, *d,* and *D_m_* values, accompanied by increased *ρ* and Rc, which markedly reduced the particle size during the transition of WMA paste into the high-SI WMA ternary complex, thereby promoting the formation of nano WMA ternary complex particles. Furthermore, stronger intermolecular interactions inside the granules caused the surface amylopectin branch clusters to contract markedly, creating high steric hindrance and reducing the Rq [15,20]. Consequently, the increased particle surface strength and reduced protruded volume of the “blocklet” enhanced the homogeneity and entropy. Taken together, these observations suggest that the hydrolytic channels became narrower, the hydrophobic effect was strengthened, and the inter- and external hardness of particles increased [7,9]. Therefore, during enzymatic digestion, the higher rigidity and re-association ability of the gel network facilitated the rapid reorganization of the fractured crystalline regions, thereby increasing the TV, FV, and SBV after the WMA ternary self-assembly [2,21]. These findings indicate that a larger proportion of SDS structures were present in the high-SI nano WMA ternary complex, promoting resistance to enzymatic penetration of glycosidic bonds [6], together with the compact and ordered “multi-phase non-periodic system” structure, leading to the continuous decline in *k*_1_, *k*_2_, *C_1∞_*, *C_2∞_*, HI, and EGI throughout the high-SI nano WMA ternary self-assembly behavior. Additionally, the SDS and RS contents of the high-SI WMA ternary complex were higher than those of hydrothermally treated bean starch reported in previous research (12.04% and 5.16%), whereas the *k*_1_ and *k*_2_ values of the WMA ternary complex were lower than those of the bean starch (7.65 and 2.70 × 10^−2^ min^−1^) [28]. Although the SDS content of the WMA ternary complex was broadly similar to that of extrusion cooking starches from staple crops (30.75–47.66%) reported previously, its higher RS content and lower *k* value compared with these staple-crop starches (1.90–29.55% and 1.43–2.06 h^−1^) resulted in a lower EGI than that of the staple-crop starches (90.34–98.93) [33]. Furthermore, the SDS (7.00–18.5%) and RS (8.86–29.3%) contents of branching-enzyme-modified maize starch pastes reported earlier were lower than those of the WMA ternary complex, whereas the corresponding k values (3.91–5.41 × 10^−2^ min^−1^) were higher [34,35]. In summary, these outcomes indicated that the highly efficient self-assembly behavior of the high-SI nano WMA ternary complex markedly slowed its digestive rate, while not rendering it completely resistant to digestion. During this gradual digestive process, the WMA ternary complex could provide a sustained release of the three essential nutrients. Thereby, high-SI nano WMA ternary complex could be presented as a highly promising novel food material with wider potential applicability across the general population.

## 4. Conclusions

In the present research, the correlation between the self-assembly behavior of high-SI nano amylopectin ternary complex and the slow digestion mechanism was systematically investigated. The results showed that ternary self-assembly characteristic peaks, accompanied by transformations in crystallinity and molecular configuration, were exhibited after the WMA ternary self-assembly. This implied the formation of the nano high-SI WMA ternary complex through non-covalent interactions. Moreover, the PCA and correlation network analyses (r > 0.99 or r < −0.99, *p* < 0.01) further confirmed that, during the transition of WMA paste to the high-SI nano WMA ternary complex, the increases in α-1,6 glycosidic linkages and CH_2_OH groups raised the SI and strengthened hydrogen bonding, electrostatic forces, and hydrophobic interactions. These effects increased the number of helical structures as well as improved rigidity and re-associate ability of chains. Such change promoted the formation of compact and ordered helical arrangements, which in turn generated a larger proportion of perfect crystalline structures. As a result, the molecular configuration and conformation shifted from a diffuse to an ordered state, the “blocklet” size changed, and the granule surface became compact and smooth. These structural changes markedly enhanced thermal resistance, molecular gel-network reformation ability, and enzymatic resistance, ultimately bringing about the increased SDS content and a decline in multi-scale digestion rate constants and EGI. Moreover, future research will focus on the effect of the SDS structure of the WMA ternary complex on in vivo insulin secretion, intestinal barrier integrity, and probiotic proliferation. In summary, the present study confirmed that the high-SI nano WMA ternary complex exhibited sufficient thermal stability and a reduced level of glycemic release, attributes that support its incorporation as a functional food additive in ultra-processed food systems aimed at reducing the risk of glucose metabolic dysregulation.

## Figures and Tables

**Table 1:** Changes in self-assembly characteristics during formation of WMA complexes.

Samples	SI (%)	Potential (mV)	Short-Range Order	Rc (%)
WMA paste	N.D.	−12.98 ± 0.80 ^d^	0.72 ± 0.02 ^d^	9.16 ± 0.22 ^d^
High SI WMA–protein complex	68.25 ± 1.45 ^c^	−8.66 ± 0.51 ^c^	0.86 ± 0.03 ^c^	12.35 ± 0.15 ^c^
High SI WMA–lipid complex	73.40 ± 0.79 ^b^	−7.06 ± 0.47 ^b^	1.05 ± 0.02 ^b^	15.71 ± 0.60 ^b^
High SI WMA–ternary complex	82.58 ± 1.17 ^a^	−5.19 ± 0.19 ^a^	1.10 ± 0.03 ^a^	23.68 ± 0.39 ^a^

WMA, white waxy maize amylopectin; SI, self-assembly index; Rc, relative crystallinity. Different superscript letters within a column indicate statistically significant differences among the samples (*p* < 0.05).

**Table 2:** Changes in helical conformation during formation of WMA complexes.

Samples	Dhelix (%)	Vp (%)	Ac (%)	CH_2_OH (%)	α-1,6 (%)	α-1,4 (%)
WMA paste	19.55 ± 0.27 ^d^	1.61 ± 0.04 ^d^	78.84 ± 0.48 ^a^	5.20 ± 0.03 ^d^	5.35 ± 0.06 ^d^	94.65 ± 0.77 ^a^
High-SI WMA–protein complex	27.17 ± 0.37 ^c^	6.11 ± 0.06 ^c^	66.72 ± 0.83 ^b^	8.61 ± 0.02 ^c^	7.51 ± 0.02 ^c^	92.49 ± 0.56 ^b^
High-SI WMA–lipid complex	33.55 ± 0.15 ^b^	8.93 ± 0.05 ^b^	57.52 ± 0.73 ^c^	11.15 ± 0.05 ^d^	10.09 ± 0.07 ^b^	89.91 ± 0.84 ^c^
High-SI WMA–ternary complex	50.20 ± 0.22 ^a^	11.14 ± 0.03 ^a^	38.66 ± 0.63 ^d^	14.36 ± 0.06 ^a^	12.34 ± 0.05 ^a^	87.66 ± 0.70 ^d^

Dhelix, double helix; Vp, V-type polymorph; Ac, amorphous content; CH_2_OH, CH_2_OH group also named free side chain; α-1,6 and α-1,4, α-1,6 and α-1,4 glycosidic bonds. Different superscript letters within a column indicate statistically significant differences among the samples (*p* < 0.05).

**Table 3:** Self-assembly characteristics and lamellar parameters of WMS paste and nanoscale ternary complexes.

Starch Sample	*d* (nm)	*d*_a_ (nm)	*d*_c_ (nm)	*α*	*D_m_*
WMA paste	10.88 ± 0.25 ^a^	5.55 ± 0.07 ^a^	5.33 ± 0.06 ^a^	−2.89 ± 0.10 ^d^	2.89 ± 0.10 ^a^
High-SI WMA–protein complex	8.83 ± 0.09 ^b^	4.26 ± 0.05 ^b^	4.57 ± 0.10 ^b^	−2.40 ± 0.03 ^c^	2.40 ± 0.03 ^b^
High-SI WMA–lipid complex	7.05 ± 0.11 ^c^	2.92 ± 0.03 ^c^	4.13 ± 0.08 ^c^	−2.01 ± 0.12 ^b^	2.01 ± 0.12 ^c^
High-SI WMA–ternary complex	3.97 ± 0.12 ^d^	1.98 ± 0.08 ^d^	1.99 ± 0.09 ^d^	−1.44 ± 0.03 ^a^	1.44 ± 0.03 ^d^

*d*, semicrystalline lamellae thickness; *d_a_*, amorphous lamellae thickness; *d_c_*, crystalline lamella thickness; *α*, mass fractal exponent; *D_m_*, mass fractal dimension. Different superscript letters within a column indicate statistically significant differences among the samples (*p* < 0.05).

**Table 4:** Changes in molecular configuration parameters during complex self-assembly.

Samples	Mn (×10^7^ Da)	Mw (×10^7^ Da)	Rg (nm)	PI	*ν*	*ρ* (g mol^−1^ nm^−3^)
WMA paste	2.11 ± 0.04 ^d^	3.78 ± 0.06 ^d^	134.55 ± 1.15 ^a^	1.79 ± 0.04 ^a^	0.18 ± 0.01 ^d^	15.51 ± 0.52 ^d^
High-SI WMA–protein complex	2.87 ± 0.03 ^c^	4.23 ± 0.07 ^c^	122.04 ± 0.95 ^b^	1.47 ± 0.05 ^b^	0.35 ± 0.02 ^c^	23.27 ± 0.48 ^c^
High-SI WMA–lipid complex	3.41 ± 0.05 ^b^	4.74 ± 0.05 ^b^	90.10 ± 1.07 ^c^	1.39 ± 0.04 ^c^	0.41 ± 0.02 ^b^	64.80 ± 0.62 ^b^
High-SI WMA–ternary complex	4.07 ± 0.03 ^a^	5.25 ± 0.08 ^a^	74.82 ± 0.44 ^d^	1.28 ± 0.05 ^d^	0.49 ± 0.01 ^a^	125.34 ± 0.22 ^a^

Mn and Mw, weight-average and number-average molecular mass; Rg, molecular gyration radius; PI, polydispersity index; *ν*, molecular conformation index; *ρ*, molecular density. Different superscript letters within a column indicate statistically significant differences among the samples (*p* < 0.05).

**Table 5:** Nano surface information of WMA paste and its ternary complex particles.

Starch Sample	Rq (nm)	Textural Features				
		Energy (×10^−3^ J)	Contrast	Homogeneity	Entropy (e.u.)	Fractal Dimension
WMA paste	17.64 ± 0.18 ^a^	1.64 ± 0.33 ^d^	0.98 ± 0.09 ^a^	0.19 ± 0.05 ^d^	9.99 ± 0.11 ^a^	6.48 ± 0.10 ^a^
WMA–lipid complex	11.41 ± 0.11 ^b^	2.05 ± 0.17 ^c^	0.78 ± 0.07 ^b^	0.28 ± 0.09 ^c^	7.90 ± 0.05 ^b^	5.16 ± 0.11 ^b^
WMA–protein complex	9.22 ± 0.07 ^c^	2.80 ± 0.09 ^b^	0.56 ± 0.03 ^c^	0.42 ± 0.10 ^b^	5.99 ± 0.08 ^c^	3.98 ± 0.14 ^c^
WMA ternary complex	6.01 ± 0.05 ^d^	3.55 ± 0.16 ^a^	0.50 ± 0.03 ^d^	0.63 ± 0.08 ^a^	4.23 ± 0.06 ^d^	2.99 ± 0.13 ^d^

Rq, root-mean-square nano roughness. Different superscript letters within a column indicate statistically significant differences among the samples (*p* < 0.05).

**Table 6:** Gelatinization characteristic parameters within the main peak during complex self-assembly.

Samples	To (°C)	Tp (°C)	Tc (°C)	R (°C)	ΔHg (J/g)
WMA paste	70.06 ± 0.66 ^d^	81.84 ± 0.75 ^d^	92.18 ± 0.25 ^d^	22.12 ± 0.41 ^d^	9.94 ± 0.17 ^d^
High SI WMA–protein complex	79.33 ± 0.77 ^c^	90.97 ± 1.21 ^c^	102.54 ± 0.61 ^c^	23.21 ± 0.16 ^c^	11.22 ± 0.09 ^c^
High SI WMA–lipid complex	81.86 ± 0.84 ^b^	95.17 ± 0.86 ^b^	106.18 ± 0.47 ^b^	24.32 ± 0.37 ^b^	15.11 ± 0.02 ^b^
High SI WMA–ternary complex	84.57 ± 0.58 ^a^	98.65 ± 0.56 ^a^	114.37 ± 0.83 ^a^	29.80 ± 0.25 ^a^	18.58 ± 0.07 ^a^

To, start temperature; Tp, gelatinization temperature; Tc, conclusion temperature; R, temperature range; ΔHg, gelatinization enthalpy. Different superscript letters within a column indicate statistically significant differences among the samples (*p* < 0.05).

**Table 8:** The digestible fractions parameters for WMA paste and nanoscale WMA ternary complexes.

Samples	RDS (%)	SDS (%)	RS (%)	HI	EGI
WMA paste	74.65 ± 0.63 ^a^	19.86 ± 0.07 ^d^	7.49 ± 1.08 ^d^	105.30 ± 0.96 ^a^	97.52 ± 0.65 ^a^
High-SI WMA–protein complex	57.17 ± 0.38 ^b^	24.10 ± 0.47 ^c^	18.73 ± 0.15 ^c^	92.47 ± 0.37 ^b^	90.47 ± 0.23 ^b^
High-SI WMA–lipid complex	43.13 ± 0.13 ^c^	28.05± 0.08 ^b^	28.82± 0.18 ^b^	83.99 ± 0.19 ^c^	85.82 ± 0.17 ^c^
High-SI WMA–ternary complex	24.48 ± 0.19 ^d^	43.28 ± 0.28 ^a^	32.24 ± 0.22 ^a^	71.83 ± 0.47 ^d^	79.15 ± 0.26 ^d^

RDS, rapidly digestible starch; SDS, slowly digestible starch; RS, resistant starch; HI, hydrolysis index; EGI, estimated glycemic index. Different superscript letters within a column indicate statistically significant differences among the samples (*p* < 0.05).

**Table 9:** In vitro LOS kinetic parameters for WMA paste and nanoscale WMA ternary complexes.

Samples	Phase I			Phase II		
	*C_1∞_* (%)	*k*_1_ (×10^−2^ min^−1^)	*t_1_* (min)	*C_2∞_* (%)	*k*_2_ (×10^−2^ min^−1^)	*t_2_* (min)
WMA paste	89.39 ± 0.48 ^a^	7.89 ± 0.05 ^a^	120 ^c^	90.13 ± 2.24 ^a^	3.20 ± 0.03 ^a^	540 ^a^
High-SI WMA–protein complex	78.49 ± 0.77 ^b^	4.79 ± 0.04 ^b^	150 ^b^	80.01 ± 1.55 ^b^	2.56 ± 0.04 ^b^	540 ^a^
High-SI WMA–lipid complex	70.95 ± 0.90 ^c^	3.68 ± 0.03 ^c^	150 ^b^	71.54 ± 1.03 ^c^	1.92 ± 0.04 ^c^	540 ^a^
High-SI WMA–ternary complex	60.21 ± 0.88 ^d^	2.43 ± 0.02 ^d^	200 ^a^	61.06 ± 0.41 ^d^	1.44 ± 0.03 ^d^	540 ^a^

*C_1∞_* and *k*_1_, equilibrium concentration and speed rate constants at first enzymolysis stage; *C_2∞_* and *k*_2_, equilibrium concentration and speed rate constants at second enzymolysis stage; *t_1_*, enzymolysis time of initial phase; *t_2_*, enzymolysis time of final phases. Different superscript letters within a column indicate statistically significant differences among the samples (*p* < 0.05).

**Figure A2:**
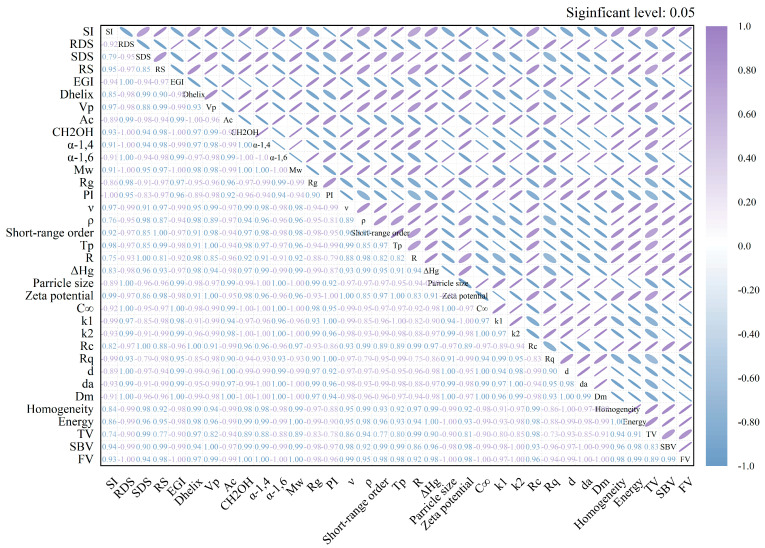
The correlation matrix dataset derived from the PCA analysis.

## Data Availability

The original contributions presented in this study are included in the article. Further inquiries can be directed to the corresponding authors.

## Abbreviations

WMA	White waxy maize amylopectin
SI	Self-assembly index
Rc	Relative crystallinity
Dhelix	Double helix
Vp	V-type polymorph
Ac	Amorphous content
CH_2_OH	Free side chain
α-1,6	α-1,6 glycosidic bonds
α-1,4	α-1,4 glycosidic bonds
*d*	Semicrystalline lamellae thickness
*d_a_*	Amorphous lamellae thickness
*d_c_*	Crystalline lamella thickness
*α*	Mass fractal exponent
*D_m_*	Mass fractal dimension
PV	Peak viscosity
TV	Trough viscosity
BDV	Breakdown viscosity
FV	Final viscosity
SBV	Setback viscosity
PT	Pasting temperature
Mn	Weight-average molecular weight
Mw	Number-average molar mass
Rg	Radius of gyration
PI	Polydispersity index (Mw/Mn)
*ν*	Conformation index
*ρ*	Molecular density
To	Start temperature
Tp	Gelatinization temperature
Tc	Conclusion temperature
R	Temperature range
ΔHg	Gelatinization enthalpy;
RDS	Rapidly digestible starch
SDS	Slowly digestible starch
RS	Resistant starch
*C*_1∞_	Equilibrium concentration at first enzymolysis stage
*k*_1_	Speed rate constants at first enzymolysis stage
*C*_2__∞_	Equilibrium concentration at second enzymolysis stage
*k*_2_	Speed rate constants at second enzymolysis stage
*t*_1_	Enzymolysis time of initial phase
*t*_2_	Enzymolysis time of final phases
HI	Hydrolysis index
EGI	Estimated glycemic index

## Appendix A

### Highlight

- High-assembly-index (SI) nano amylopectin (WMA) ternary complex was novelly prepared.
- Crystal type and conformation were orderly changed during high-SI nano WMA assembly.
- Non-covalent forces led to a rising assembly site and free side chain upon assembly.
- Reducing enzymatic channel sizes and more compact granules were found after assembly.
- Rising slowly digestible starch led to more stable glycemic release during assembly.

## Appendix B

**Table A1:** In vitro traditional kinetic parameters for WMA paste and nanoscale WMA ternary complexes.

Samples	*C*_∞_ (%)	*k* (×10^−2^ min^−1^)	HI	EGI
WMA paste	89.94 ± 1.14 ^a^	5.70 ± 0.05 ^a^	104.99 ± 1.10 ^a^	97.36 ± 0.77 ^a^
High-SI WMA–protein complex	80.85 ± 2.00 ^b^	3.72 ± 0.04 ^b^	92.79 ± 0.66 ^b^	90.81 ± 0.52 ^b^
High-SI WMA–lipid complex	71.75 ± 1.66 ^c^	2.88 ± 0.06 ^c^	84.21 ± 0.85 ^c^	86.03 ± 0.28 ^c^
High-SI WMA–ternary complex	59.98 ± 1.02 ^d^	1.77 ± 0.03 ^d^	71.44 ± 1.18 ^d^	78.06 ± 0.29 ^d^

Different superscript letters within a column indicate statistically significant differences among the samples (*p* < 0.05).
